# The EMO-Model: An Agent-Based Model of Primate Social Behavior Regulated by Two Emotional Dimensions, Anxiety-FEAR and Satisfaction-LIKE

**DOI:** 10.1371/journal.pone.0087955

**Published:** 2014-02-04

**Authors:** Ellen Evers, Han de Vries, Berry M. Spruijt, Elisabeth H. M. Sterck

**Affiliations:** 1 Animal Ecology, Utrecht University, Utrecht, the Netherlands; 2 Ethology Research, Biomedical Primate Research Center, Rijswijk, the Netherlands; Federal University of Rio Grande do Norte, Brazil

## Abstract

Agent-based models provide a promising tool to investigate the relationship between individuals’ behavior and emerging group-level patterns. An individual’s behavior may be regulated by its emotional state and its interaction history with specific individuals. Emotional bookkeeping is a candidate mechanism to keep track of received benefits from specific individuals without requiring high cognitive abilities. However, how this mechanism may work is difficult to study in real animals, due to the complexity of primate social life. To explore this theoretically, we introduce an agent-based model, dubbed EMO-model, in which we implemented emotional bookkeeping. In this model the social behaviors of primate-like individuals are regulated by emotional processes along two dimensions. An individual’s emotional state is described by an aversive and a pleasant dimension (anxiety and satisfaction) and by its activating quality (arousal). Social behaviors affect the individuals’ emotional state. To implement emotional bookkeeping, the receiver of grooming assigns an accumulated affiliative attitude (LIKE) to the groomer. Fixed partner-specific agonistic attitudes (FEAR) reflect the stable dominance relations between group members. While the emotional state affects an individual’s general probability of executing certain behaviors, LIKE and FEAR affect the individual’s partner-specific behavioral probabilities. In this way, emotional processes regulate both spontaneous behaviors and appropriate responses to received behaviors, while emotional bookkeeping via LIKE attitudes regulates the development and maintenance of affiliative relations. Using an array of empirical data, the model processes were substantiated and the emerging model patterns were partially validated. The EMO-model offers a framework to investigate the emotional bookkeeping hypothesis theoretically and pinpoints gaps that need to be investigated empirically.

## Introduction

Many studies on animal behavior have contributed to a comprehensive body of knowledge concerning specific emotions (such as anxiety) and the relation between the underlying neurobiology and behavior. Yet, our understanding of how emotional processes within individuals regulate their behavior and how this in turn may result in group-level patterns, such as partner-specific reciprocity or the development and maintenance of affiliative relationships, is poor. To empirically study this, i.e. by manipulating distinctive factors within one or a few group members and assessing the resulting group-level changes, is methodologically difficult. Such invasive experiments may affect also other behaviors and regulatory processes and, furthermore, may disturb the complex relations and behavioral processes within the group. Observational studies of naturally occurring stressful events [1] may give valuable insights. Yet, it remains difficult to untangle the exact causalities underlying the observed changes at the group level, as social behavior comprises many interactions of many individuals. Here, we present an agent-based model (ABM), providing a (biologically relevant) alternative tool to allow exploratory research on the patterns of social relationships and the necessary, underlying requirements. We developed our model, dubbed the *EMO-model*, using empirical data to substantiate the implemented emotional processes and behavioral rules. In this introduction, we first review the empirical and theoretical background on emotional processes and their role in regulating social behavior, specifically in macaques. We continue with the theoretical background on emotional bookkeeping and, finally, we describe the status quo of our specific contribution to agent-based models of social species, such as macaques.

### Two Dimensions of the Emotional State: Anxiety and Satisfaction

Emotional processes are considered a prompt response to a (social) event preparing an individual to quickly react in a certain direction. The response consists of activating a concerted set of actions at the physiological (peripheral and central) and behavioral level [2], comprising the interface between perception, a first fast analysis of sensory input, and subsequent behavioral reaction [3]. Emotional processes motivate an individual to conduct a certain behavior efficiently adapted to meet challenges of the (social) environment [4], [5]. In this paper, we refer to emotional processes and emotional states without implying accompanying conscious, subjective states that are inaccessible to measurement in non-human animals [2], [4], [6], [7].

The quality component of an individual’s emotional state, often referred to as valence or appraisal, has been proposed to comprise an aversive and a pleasant dimension [8], [9]. Social behavior of group living animals may also be described in terms of this dichotomy, encompassing an agonistic and affiliative dimension, which relate to different aspects of social organization, such as dominance hierarchies and affiliative bonds. Moreover, emotional processes employ different underlying mechanisms, as aversive emotional responses (e.g. anxiety, stress) are more associated with the amygdala and the HPA axis [10], [11], while pleasant emotional responses employ the reward system consisting mainly of the ventral tegmental area (VTA), the opioid-dopamine system and oxytocin [12]. This suggests that social behavior may be regulated via two underlying emotional dimensions that relate to agonistic and affiliative behavior, respectively. These two emotional dimensions are, hereafter, referred to as anxiety (aversive) and satisfaction (pleasant). Anxiety and satisfaction are assumed to be independently regulated, e.g. a decrease in anxiety does not automatically result in increased satisfaction.

Anxiety can be defined as a state of apprehension or uneasiness that stems from (anticipation of) danger [13]. In contrast to fear, which is directed at a specific stimulus (object or subject) [14], anxiety may be seen as a state of general ‘fearfulness’, in response to negative stimuli within the recent and current (social) environment as perceived by the individual. In parallel, satisfaction may be defined as a state of general ‘contentedness’, in response to positive stimuli within the recent and current (social) environment as perceived by the individual.

Emotional processes have been proposed to mediate between social interactions and the behavioral and physiological response [3], [15]–[18]. For example in macaques, upon aggression, the recipient as well as the aggressor will show increased levels of anxiety [18]–[20], which may enhance the individuals’ tendency to affiliate [18]. Affiliation will in turn decrease anxiety [19] and may increase satisfaction. In macaques and other primates, it has been shown, that grooming is pleasant for the receiver, but also the actor [21]–[23].

Moreover, primates use communicative signals (i.e. facial expressions) to express their behavioral intentions towards group members, thereby affecting the behavior of (potential) interaction partners. For instance in macaques, affiliative and submissive signals (such as ‘lip-smacking’ or ‘bared-teeth’) are thought to appease the receiver of the signal, thereby reducing the risk of aggression or facilitating affiliation [24]–[26], while aggressive signals (such as ‘open-mouth threat’) may result in avoidance or counter-aggression [27], [28]. As such, the expression and perception of communicative signals may also employ underlying emotional processes [29]. In this way, emotional processes may be involved in a homeostatic mechanism regulating spontaneous behaviors as well as appropriate responses to received behaviors on a short-term basis.

### Activating Quality of the Emotional State: Arousal

An individual’s emotional state cannot only be characterized by its valence (anxiety and satisfaction), but also by its arousal [2], [14], [30]. Arousal refers to the intensity of an emotional state or its activating quality [2], [31], which may depend on the salience or relevance of the stimulus that elicited the specific emotional state. Arousal has been defined as an individual’s level of sensory-motor responsiveness, its (physiological) activity or its motivational state [32]–[34]. In ethological research, several physiological parameters and displacement behaviors (as put forward by [35]) (e.g. heart rate and self-scratching) are commonly used as indicators of emotional arousal in primates (macaques: [36]–[38], squirrel monkeys: [33], capuchins: [39], baboons: [40], chimpanzees: [41], and also in greylag geese [42]). For instance in macaques, scratching and heart rate have been observed to increase quickly upon stressful (social) events, such as (the risk of) aggression [16], [20], [36], [43] and to decrease afterwards within several minutes [22], [44]. Positive (social) events, such as grooming, have been shown to facilitate an even faster decrease of these measures [22], [43]. Thus, arousal levels may change in response to (social) stimuli and correspond with the individual’s state of responsiveness, activity or inclination to act. In macaques, increased vigilance behavior and motor activity are commonly associated with increased arousal [33]. As high arousal levels are often caused by received aggression, the resulting increase of (social) vigilance may serve to avoid further potential aggression or to find potential affiliation partners.

### Emotional Bookkeeping via Partner-specific Emotional Attitudes

Emotional processes have also been proposed to mediate the development, maintenance and assessment of social relations [15]–[17], [45]. Aureli and colleagues have developed a hypothetical framework of emotional bookkeeping as a mediator of primate social relationships [15], [17], [45]. While recent social interactions affect an individual’s (general) emotional state and, thus, its general behavior, individuals may also integrate the emotional states that accompanied earlier social interactions into an emotional attitude that is associated with each respective interaction partner. This partner-specific emotional attitude may in turn affect an individual’s subsequent behavior towards the respective partner. In this way, fleeting emotional responses may be accumulated using a simple partner-specific bookkeeping mechanism, similar to that underlying de Waal’s concept of ‘attitudinal reciprocity’ [46]. In contrast to ‘attitudinal reciprocity’ [46] and the individual’s general emotional state, both of which are assumed to work on a short-term scale, emotional bookkeeping has been proposed to integrate information over longer periods and, thus, to affect partner-specific behavior on a longer term [45].

Since an individual’s general emotional state is not specific to interaction partners, it constitutes a cognitively simpler mechanism than emotional bookkeeping, which is based on the rate and intensity of the emotional responses to the interactions with a particular individual and, thus, does require individual recognition. However, emotional bookkeeping does not require a specific memory of who did what and when (‘episodic-like memory’: [47]), due to the conversion of earlier interactions into a common currency, i.e. the partner-specific emotional attitude [45]. In other words, different types of social interactions (e.g. affiliation and support) with different partners may result in qualitatively similar partner-specific attitudes, resulting in qualitatively similar behavioral responses. However, the emotional attitudes assigned to several partners may differ quantitatively, for example between a frequent and an occasional groomer. As such, emotional bookkeeping may allow for differentiated valuation of partners dependent on quality and quantity of earlier interactions with these partners.

In line with the two dimensions of the general emotional state, anxiety and satisfaction, we here propose that a partner-specific emotional attitude also comprises an agonism-related and an affiliation-related dimension. Hereafter, these will be referred to as FEAR and LIKE attitude, respectively.

In the EMO-model, LIKE attitudes are implemented as the partner-specific equivalent of satisfaction. While an individual’s satisfaction describes its general ‘contentedness’, LIKE attitudes reflect an individual’s differential valuation of each group member, concerning the received affiliation over a certain timeframe. In turn, the LIKE attitude that an individual associates with a certain group member affects its probability to direct affiliative behavior towards this group member. In this way, LIKE attitudes may regulate the development and maintenance of partner-specific affiliative relations.

FEAR attitudes are implemented in the EMO-model as the partner-specific equivalent of anxiety and describe the agonistic relationships between an individual and each other group member, as perceived by the individual. Group members from whom an individual receives a lot of aggression cause high anxiety levels in this individual. Thus, the individual is expected to assign high FEAR attitudes to such group members. In this way, FEAR attitudes can be seen as an internal representation of the dominance relations with the group members. Such partner-specific bookkeeping of dominance outcomes has previously been proposed and implemented in agent-based models [48], [49]. However, in many primates, dominance hierarchies are stable over long periods of time (up to several years, macaques: [50]–[53], gorilla: [54], baboons: [55], [56], capuchins: [57], vervets: [58]). As we do not aim to study the development of a dominance hierarchy, but want to focus on the development and specifically the maintenance of affiliative relationships, we assume that in our model group a dominance hierarchy has been already established and does not change over the timeframe of two years in our model simulation. Therefore, FEAR attitudes in the EMO-model do not change dynamically over time and simply resemble the fixed rank distance between two individuals. In turn, FEAR attitudes affect the probability to direct submissive or aggressive behavior towards this partner. Assuming a fixed hierarchy and fixed FEAR attitudes, while studying dynamic LIKE attitudes seems reasonable as a starting point. In a study on macaques, where unfamiliar individuals were paired, unresolved dominance relations prevented engagement in affiliation [59], and thus also the potential development of LIKE attitudes.

### Additions to Existing ABM of Primate Social Behavior

ABMs are a powerful tool to study social behavior, as they can reveal the potential for self-organization in social systems, showing that simple behavioral rules may lead to complex patterns at the group level, in primates [49], [60]–[68] and other species (birds: [69], fish: [70]–[73], insects: [74]–[78]). For instance, ABM studies have demonstrated that spatial centrality of dominants may emerge from minimalistic behavioral rules concerning dominance interactions [49], [74]. In one of these models, DOMworld [49], the most extensively published ABM on primate behavior, low-ranking individuals typically lose dominance interactions and subsequently flee from their opponent. The resulting spatial group structure in turn results in differentiated interaction frequencies between different group members, potentially regulating social group properties (such as the dominance hierarchy). These outcomes indicate the potential of self-organization in real animals. However, the full validity of DOMworld has been questioned [79], since real primates also employ other behaviors to regulate aggression, e.g. avoidance of the aggressor or monitoring its behavior and location. Implementing such alternative behavioral rules in an ABM, we have demonstrated elsewhere that different sets of behavioral rules concerning movement and perception may result in a similar spatial group structure, but may differ in other properties, such as the frequencies and patterns of interactions [66], [67]. This stressed the importance of implementing multiple levels of a system into a model of this system [80] to subsequently substantiate and validate patterns on multiple levels of the system [81].

Most ABMs on primate behavior mentioned above did not include an emotional valuation of social behavior and only concerned one dimension of social behavior, i.e. aggression-submission. More recently, in the GrooFi-world model, an extended version of the DOMworld model, also an affiliative behavior (grooming) has been implemented [65], [68]. Moreover, a first attempt has been made to complement the behavioral rules with an underlying emotional component, namely anxiety. This model generates interesting behavioral patterns, such as reconciliation, grooming after a fight, grooming up the hierarchy and coalitions.

Here, we present a new ABM, the EMO-model, in which we explicitly incorporated the interrelation between social behavior and emotional states of primates along two dimensions (see above). The agonism-related dimension concerns agonistic behavior and anxiety and shares many features with previous models. The affiliation-related dimension concerns grooming and satisfaction. For the agonism-related dimension, many general features of our model resemble the GrooFi-world model. In both models, aggression increases anxiety, which subsequently enhances the tendency to groom and in turn decreases anxiety. However, in the GrooFi-world model emotional processes only involve anxiety. For instance, lack of grooming was implemented to result in high anxiety, subsequently enhancing the tendency to groom. In contrast, our EMO-model distinguishes between anxiety, satisfaction and arousal, backed by empirical data. Here, lack of grooming was implemented to result in low satisfaction levels and slightly increased arousal. As a result, the tendency to groom and the tendency to engage in active behavior as opposed to resting are enhanced. So, grooming tendency is enhanced through high anxiety levels (due to aggression) and low satisfaction levels (due to lack of grooming). The effect of satisfaction on grooming constitutes the affiliation-related dimension.

Furthermore, aggressive behavior in both models is risk-sensitive. However, while this risk-sensitivity only depends on the dominance relation between two opponents in GrooFi-world, it is additionally dependent on the actor’s anxiety levels in the EMO-model. Moreover, to our knowledge our model is the first agent-based model of primates that also implements partner-specific emotional attitudes along two dimensions, i.e. not only FEAR but also LIKE attitudes. Thus, this paper presents a first attempt to explicitly implement the process of emotional bookkeeping into an agent-based model of primate social behavior.

In contrast to many earlier models on primate social behavior [49], [65], [68], and building up on earlier attempts [66], [67], here we explicitly define not only the aggressive, but also the submissive components of agonism, i.e. fleeing, submissive signaling and avoidance. While unidirectional signals of submission are not observed in very egalitarian species [82]–[84], most macaque species direct submissive signals exclusively at higher-ranking individuals [85]–[87]. For this reason, submissive signals are commonly used to determine the hierarchy within a group. In our model, submission was implemented such that it was exclusively directed at individuals that were higher in rank. Thus, we model a unidirectional, linear hierarchy. To our knowledge, this model is also the first agent-based model on primate social behavior that explicitly implemented communicative signaling. We implemented affiliative, submissive and aggressive signals that may represent ‘lip-smack’, ‘bared-teeth’ and ‘open-mouth threat’ in macaques. In the EMO-model, physical interactions, e.g. grooming and attacking, are only executed when in close proximity (1 m) to the interaction partner. The choice of such a distance for physical interactions is logical, but in contrast to other ABM on primate behavior. For instance in GrooFi-world, individuals can be groomed at a distance up to 8 units [65]. Furuichi found that in a troop of Japanese macaques, aggression occurred mostly when the two opponents were closer to each other than 1 m and only rarely when the distance was larger [88].

Agent-based modeling permits a stronger coupling between the two phases in scientific research: “obtaining empirical data” and “constructing an explanatory theoretical model” [75], since ABM help to determine crucial parameters for explanatory models and reveal which empirical data are still missing. Accordingly, we aimed to choose the parameter settings of our model as realistic as possible, promoting the exchange between empirical data and models of behavior [89]. To do this, we first surveyed empirical data of macaques to obtain an overview of realistic margins of general behavioral frequencies. We then tuned the general probabilities of executing these behaviors in our model, such that the resulting average behavioral frequencies were representing the empirical data well. Subsequently, to validate the EMO-model, we assessed whether our model was also able to reproduce patterns from empirical macaque data on a group or subgroup level. Such higher-level patterns are not explicitly implemented into the model, but emerge from the interactions of the model entities. This feature of ABM allows investigation of the link between emotional processes and patterns in social behavior at the group level.

In sum, we explicitly incorporated the proposed interrelations between primate social behavior and emotional processes, instead of modeling this as a black box. This way, the EMO-model serves as an explicitly formulated hypothesis, about how emotional processes may regulate primate social behavior and vice versa. Following Petit [89], this model integrates several partial hypotheses (on emotional processes and emotional book-keeping in animals) and the current state of empirical data, which complement each other. We aimed to produce a representative model that may also generate structural properties at a group level. This model allows us to pinpoint gaps in the empirical data and in the theoretical understanding of the underlying mechanism. Our model serves as an initial attempt to study the emotional regulation of social processes and may offer a promising framework to study the complex dynamics of social relationships.

In this paper, we further describe the implementation of the model processes in detail, following the ODD protocol [90]. Next, we provide the validation of the EMO-model on multiple levels and present the general patterns that emerge in the model. Finally, we discuss the significance of the EMO-model to study complex behavioral patterns of social species such as macaques.

## Methods

Simulations were run using NetLogo 5.0.2 [91]. The program code of all models will become available via the *Publications* website of the *Animal Ecology* group (http://www.uu.nl/faculty/science/EN/contact/depts/biology/research/chairs/bb/Publications/Pages/default.aspx). Below, we describe our models according to the updated ODD protocol [90]. This protocol is a standardized method of describing agent-based models, which ensures the model description to be more complete and better comparable to other models, allowing also reproducibility of the model. Following the ODD protocol (Overview, Design concepts, Details), this Methods section is structured as follows. The *‘Overview’* part presents the general description of the model’s 1) Purpose, its 2) Entities, state variables and scales and the 3) Process overview and scheduling. The *‘Design concepts’* part describes ABM-specific characteristics and concepts of the model, namely 4) Basic principles, 5) Emergence, 6) Adaptation, 7) Learning, 8) Sensing, 9) Interactions, 10) Stochasticity and 11) Observation. The *‘Details’* part covers the 12) Initialization of the model, as well as the detailed implementation of parts and processes in the model, which are referred to as 13) Submodels. Additionally, this Methods section includes a subsection on the 14) Simulation experiments and the 15) Statistical analysis, which are not part of the ODD protocol.

### 1. Purpose

This model serves as an initial attempt of an explicitly formulated hypothesis, about how emotional processes may regulate primate social behavior and vice versa. We aimed to develop a model that a) reproduces general patterns of primate behavior and b) may also generate more complex patterns of behavior similar to those observed in primate groups. We analyze the effect of the degree to which emotional bookkeeping affects affiliative partner choice, on the general behavioral patterns within the model.

### 2. Entities, State Variables and Scales

We simulated the movements and interactions of 20 primate-like model individuals. These individuals are characterized by a number of state variables, which are summarized in Table S1 and described hereafter.

Individuals are characterized by their dominance strength (myDOM), which ranges from *1/N* (for the lowest-ranking individual) to *1.0* (highest-ranking), where N describes the total number of animals in the group. Dominance strength does not change over time or after interactions [66], [67], [79]. Individuals also differ in their (current) schedule time, myTIME, which determines when the individual is activated anew. Furthermore, individuals differ in their (current) scanning probability, myPscan, and the related variable myVIEW_ANGLE (the current width of the view angle). The variables myTIME, myPscan, myVIEW_ANGLE and the spatial coordinates of the individuals change dynamically over the course of the whole simulation.

Next to these general state variables, our model entities are further described by their emotional state, consisting of an aversive and a pleasant dimension (myANXIETY and mySATISFACTION) and myAROUSAL, an individual’s state of alertness or responsiveness to stimuli. While arousal affects an individual’s general probability to engage in active behavior (in response to stimuli), both, anxiety and satisfaction enhance appropriate and inhibit inappropriate responses to stimuli. Arousal, anxiety and satisfaction may change dynamically over time depending on the social context of ego (i.e. a model entity). The current level of arousal, anxiety and satisfaction that is approached over time (myAROUSAL_LIMIT, myANXIETY_LIMIT, mySATISFACTION_LIMIT) depending on the currently experienced social context of the individual also changes dynamically over time. For instance during grooming satisfaction level slowly approaches 1.0, while in the absence of grooming it slowly approaches 0.0. Besides the general emotional state, individuals are also characterized by partner-specific emotional attitudes (LIKE and FEAR) they assign to each other group member. In our model FEAR attitudes are fixed, while LIKE attitudes are dynamically changing over time depending on earlier affiliative interactions.

General model parameters are summarized in Table S2 and described hereafter. The modeled environment is a continuous two-dimensional grid (300×300 grid units) with a torus shape to exclude disturbing border effects. The length of one grid unit resembles one meter. We did not explicitly implement ecological features of the environment; in the model an individual’s environment is purely social. This also implies that the model individuals do not engage in foraging behavior. Thus, we model a group that is not travelling.

One time step in the simulation resembles 1 MINUTE. One HOUR consists of 60 MINUTES and we defined 12 HOURS as one DAY, as this approximately resembles the active, non-sleeping part of the day for many primate species [92]. Furthermore, we defined 7 DAYS as 1 WEEK and 50 WEEKS were defined as 1 YEAR. Simulations were run for 504.000 time steps, i.e. 2 YEARS, plus a prior stabilization period of 21.600 time steps, i.e. ca. 4 WEEKS.

### 3. Process Overview and Scheduling

**Figure 1:**
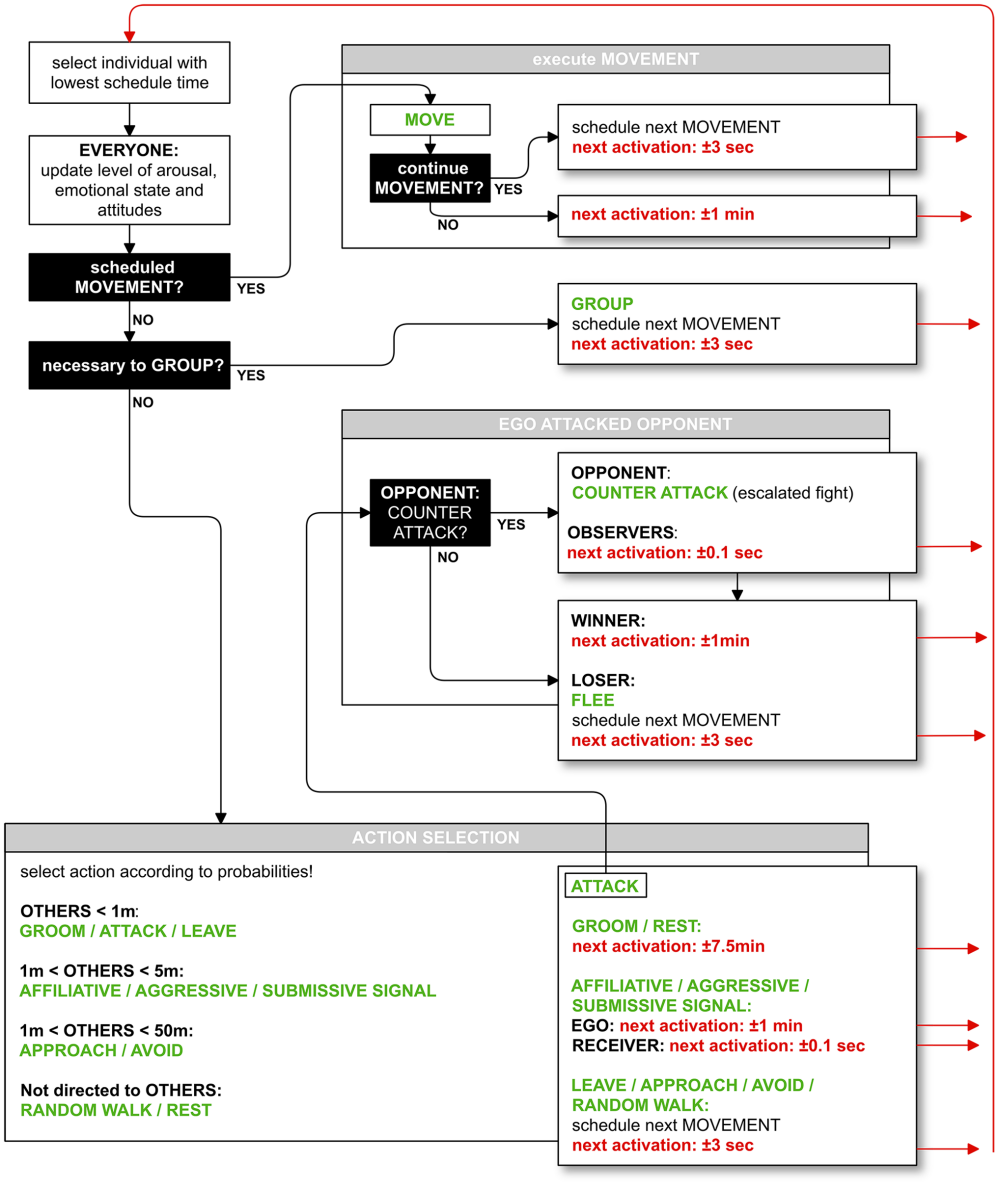
Process overview of the model. This figure illustrates the order of the processes executed by the model entities and their timing regime.

Our model is event-driven. While most social behaviors are discrete events in time, moving, resting and grooming are modeled as continuous duration behaviors. Therefore, time is modeled on a continuous scale. During a simulation run, individuals’ activations are regulated by a timing regime. The general process overview and the timing regime are illustrated in Figure 1.

Each time, the agent with the lowest schedule time is activated first. Whenever an individual is activated, first all model entities update those state variables that may have increased or decreased over the time interval that has passed since the last activation of an entity (arousal, anxiety, satisfaction, LIKE attitudes) (see *Submodels Arousal, Anxiety, Satisfaction and LIKE Attitudes* below for more details). If the activated individual had scheduled a movement action, that action is executed (see *Submodel Movement* below). Else, ego checks the grouping criteria and employs grouping, if necessary (see *Submodel Grouping* below). If no grouping and no movement are to be performed, ego may select either a social behavior, or resting or random movement within the group. Which behavior (and which interaction partner) gets selected depends on ego’s own emotional state and its arousal, as well as on its emotional attitudes towards the potential interaction partners (see *Submodel Action Selection* below). Moreover, the selected behavior may affect emotional attitudes of involved individuals. It may also affect the emotional state of ego and involved individuals (not depicted in Figure 1), as well as their schedule time (see Figure 1).

Thus, after activation, the next activation of ego, but also that of interaction partners or bystanders is scheduled anew. The exact time until an individual’s next activation depends on the behavior performed, received or observed, respectively (see *Design Concept: Stochasticity* for the random drawing of the schedule times). Movement, resting and grooming are implemented as duration behaviors and are performed in bouts. Here, after starting a movement, resting or grooming bout, ego is activated after some time to decide whether the behavior is to be continued (Table S3). As social interactions may involve (and therefore activate) other group members, they may also interrupt a grooming or resting bout. As such, whenever ego receives an attack, it is immediately activated to respond with either fleeing or a counter-attack (Table S3). Whenever ego receives a communicative signal (e.g. an aggressive signal) or observes an attack nearby, a fast reaction is required and ego is activated shortly after to select an action (Table S3).

### 4. Basic Principles

**Figure 2:**
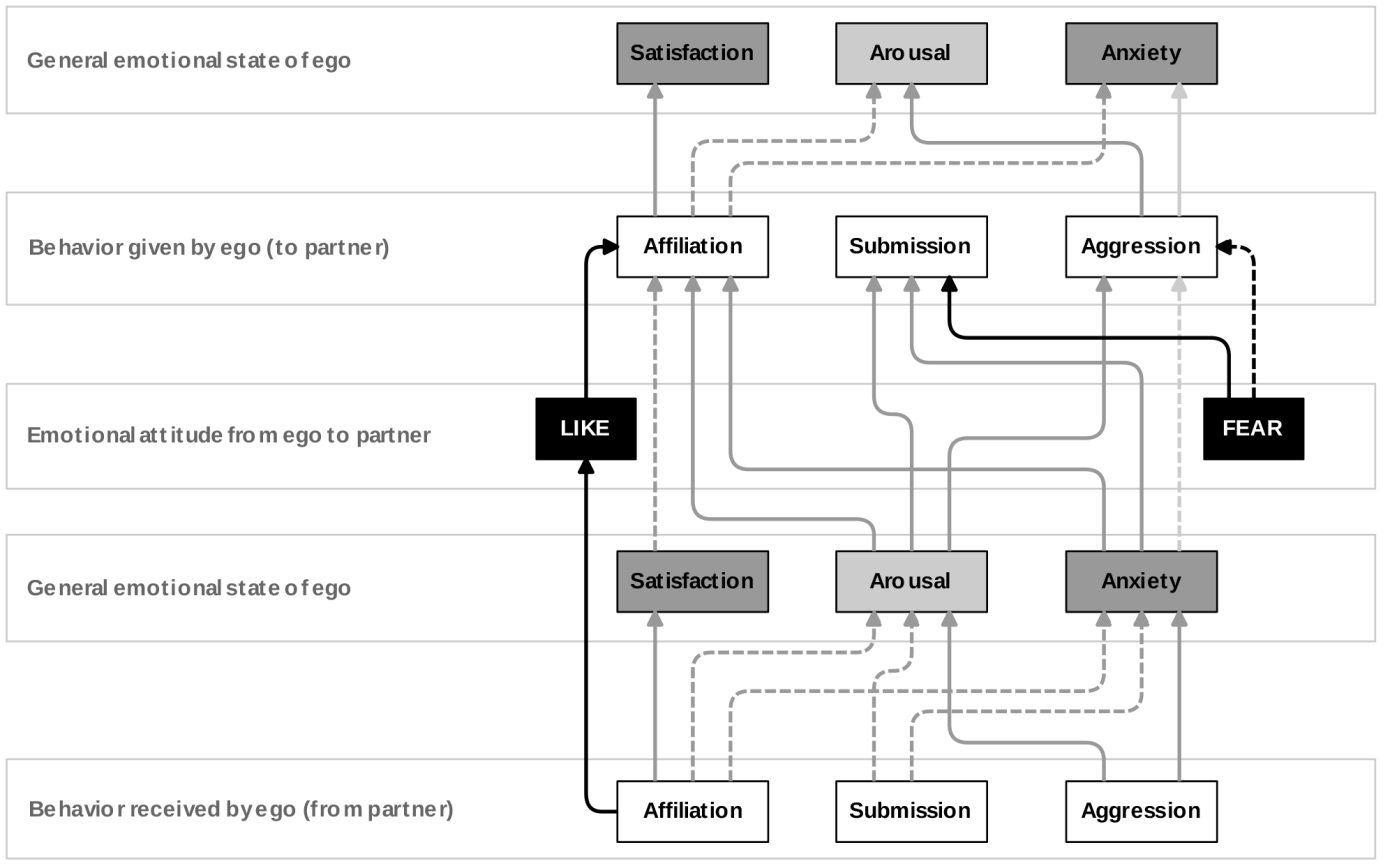
Interactions between behavior, emotional state and attitudes. This figure illustrates the effect of behavior on an individual’s emotional state and its partner-specific attitudes towards others and vice versa. Solid arrows indicate an increasing effect, while dashed arrows indicate a decreasing effect. Partner-specific effects are depicted as black and general effects are depicted as grey arrows. Light grey arrows depict effects that also depend on other factors, such as the rank of the opponent or the outcome of a fight. See *Subsection Basic Principles* and the respective *Submodels* for more details.

In our model, social behaviors affect the emotional states and attitudes of individuals. In turn, emotional state and attitudes affect the behavior of individuals. An overview of the interactions between behavior, emotions and attitudes is given below and depicted in Figure 2.

Receiving affiliative behavior increases satisfaction levels and decreases arousal and anxiety. While receiving submissive behaviors results in decreased arousal and anxiety, receiving aggressive behavior or observing (the risk of) aggression nearby results in increased levels of anxiety and arousal.

Arousal affects ego’s activity. Therefore increased arousal results in a higher general probability for all social behaviors. High satisfaction levels decrease the probability of (further) affiliation. High anxiety levels result in increased probabilities for affiliative or submissive behavior and in more risk-sensitive aggression probabilities, i.e. the aggression probability decreases, but increases towards very low-ranking individuals.

Receiving affiliation from a specific individual increases ego’s LIKE attitude towards this individual. In turn, ego’s probability to affiliate with this specific individual increases. Ego’s FEAR attitude towards a specific individual represents the rank distance between ego and this individual. FEAR is not affected by behavior, but only regulates ego’s agonistic behavior towards the specific individual: a high FEAR attitude results in high submission probabilities and low aggression probabilities.

Finally, executing affiliative behavior (i.e. grooming) increases also ego’s own satisfaction level and decreases ego’s anxiety and arousal. On the other hand, executing aggressive behavior increases ego’s own arousal. Depending on whether ego is the winner or loser of a fight, executing aggression may decrease or increase ego’s own anxiety levels.

In sum, the emotional state regulates appropriate behavior in response to received behaviors, while partner-specific emotional attitudes regulate appropriate behavior in response to specific individuals (see Figure 2).

### 5. Emergence

In agent-based models, group-level properties are usually not implemented explicitly into the model, but rather emerge from the interactions of the lower-level entities, i.e. the individuals.

Individuals in our model prefer to stay near the group, but avoid proximity to those individuals that may pose a high risk of aggression. However, the probability of avoiding such a group member also depends on ego’s own emotional state, which in turn depends on recent behaviors performed, received or observed by ego. Moreover, individuals in our model prefer to direct affiliative behavior to individuals towards whom they assign a high affiliative attitude (LIKE attitude), which in turn depends on earlier received affiliation from these individuals. Therefore, in our model, patterns of avoidance, approach and proximity are emergent properties arising from the interactions of the model entities.

As social interactions depend on spatial proximity between interaction partners and also feed back on the spatial proximity, the patterns of those social interactions are also an emergent property in our model.

LIKE attitudes in our model develop quickly, depending on earlier received affiliation from other individuals, but need to be maintained by affiliative behavior on a regular basis. LIKE attitudes affect affiliative partner choice and affiliative behaviors feed back on the level of LIKE attitudes. Therefore, in our model the structure of the network of LIKE attitudes and group level patterns such as reciprocity are emergent properties arising from the interrelation between LIKE attitudes and affiliative behavior.

Thus, while individual behavior is explicitly implemented in the model rules, resulting group level patterns of proximity, behaviors and relations are emerging from the complex interactions between the model entities.

### 6. Adaptation

The model entities change their behavior in response to changes in their general emotional state (arousal, anxiety and satisfaction) and their partner-specific emotional attitudes towards others. We assume that individuals (implicitly) seek to increase satisfaction and to decrease anxiety. As appropriate behavior is mediated by emotional processes this yields a homeostatic regulation system. In this way, we aimed to produce adaptive (in the sense of flexible) behavior and emerging group properties that are representative of observed social behavior of real primates. Note however, that the model entities do not explicitly seek to increase certain fitness measures, but simply follow rules that are expected to result in adaptive behavior.

### 7. Learning

Some traits of our model entities change in response to earlier social interactions, i.e. the emotional state (arousal, anxiety and satisfaction) and LIKE attitudes. The change in LIKE attitudes, which are partner-specific, may be seen as a (basic) form of learning. Individuals in our model regularly update their LIKE attitudes to other group members, based on earlier grooming received from these individuals. When ego receives no affiliation from a certain group member over some time, its LIKE attitude towards this individual will slowly decrease, while it may increase again after episodes of received grooming. Moreover, the LIKE attitude integrates partner-specific information about the duration and frequency of recent and earlier received grooming. This mechanism of emotional bookkeeping provides individuals with summarized information on ‘valuable’ affiliation partners, which may dynamically change over time according to these partners’ behavior. In this way, individuals ‘learn’ with which specific partners they should affiliate.

### 8. Sensing

Individuals in our model may perceive the location, certain behaviors and signals of other group members, but only locally within certain distances and within a specific view angle. The exact distances and view angles depend on the salience of the perceivable information, e.g. the presence of group members can be perceived at greater distance than the behavior of these individuals and nearby escalated fights can be perceived even when the opponents are located outside of the perceivers view angle (see *Submodel Perception and Signaling* for more details). Individuals ‘know’, are able to perceive, or are assumed to have learned the dominance strength of other group members. Perception of a group member elicits ego’s internal valuation of this group member, i.e. its FEAR and LIKE attitude that it assigned to this specific individual.

### 9. Interactions

Social interactions in our model can be categorized as affiliative (grooming, affiliative signaling and approaching), submissive (leaving, submissive signaling and avoiding) and aggressive (attacking and aggressive signaling) behaviors.

Before deciding on which behavior to perform, the potential interaction partners, i.e. the 10 nearest recognizable individuals (within MAX_DIST and ego’s current view angle) are determined. If in total less than 10 recognizable individuals are perceived by ego, then only those group members are taken into account. The decision may further depend on ego’s attitude (FEAR and LIKE) towards these individuals and ego’s general emotional state (arousal, anxiety and satisfaction). The possible behaviors towards each of these 10 (or less) nearest neighbors are each assigned a probability and one behavior (towards one specific interaction partner) is chosen randomly, according to these probabilities (see *Submodel Action Selection*).

Which social interactions can be performed towards a group member depends on the distance towards this individual (see Table S4 for an overview). Individuals within INTERACT_DIST (1 m) can be groomed, left or attacked. Individuals within PERS_DIST (5 m) can receive affiliative, submissive or aggressive signals. Individuals within MAX_DIST (50 m) can be approached and individuals within PERS_DIST (5 m) can be avoided. Note that individuals within a distance of 1 m (INTERACT_DIST) are not approached anew, as they are already in close proximity. Individuals in our model are not able to perform grooming, attack or to send signals towards an individual that is currently executing any movement behavior. However, individuals are still able to move towards or away from such an individual.

### 10. Stochasticity

In our model, many processes are not implemented deterministically, but include some degree of stochasticity, to produce variability in those processes.

When ego is to perform a behavior, each potential behavior is associated with a certain probability, depending on ego’s emotional state and its emotional attitudes towards the potential interaction partners. During action selection, one of the possible behaviors is randomly selected according to the probabilities.

When two individuals engage in an escalated fight (i.e. an attack is followed by a counter-attack), the winner is stochastically determined, depending on its win chance *w_ij_* (see *Submodel Counter-attack and escalated fight*). A higher difference in dominance strength results in a higher win chance for the dominant individual.

When executing a random walk, an individual simply moves forward for one step. Before each subsequent step, either a new orientation is chosen randomly or the same orientation is kept. Both options have a probability of 0.5.

When ego’s next activation is scheduled, the actual schedule time is drawn from a normal distribution around the appropriate mean value (depending on the context, see Table S3) with a standard deviation (SD) of 5%.

### 11. Observation

For the analysis of our model, we only used data that were recorded during the last 252.000 time steps of each simulation run, i.e. the last YEAR.

The individuals’ level of arousal, anxiety and satisfaction, the level of the dyadic LIKE attitudes and the dyadic proximity scores, were sampled every 3.5 DAYS and then averaged (per individual or dyad, respectively) over one YEAR for each simulation run. The number or duration of dyadic behaviors was recorded per dyad per behavior over each recording interval of 3.5 DAYS and then divided by the duration of the recording interval to obtain average hourly behavioral rates.

The percentage of time spent grooming was recorded per individual and was defined as the total duration of an individual’s grooming bouts divided by the total recording time, i.e. one YEAR. The exact durations of all grooming bouts were recorded per individual. The duration of a grooming bout was defined as the time interval an individual continuously engaged in grooming, i.e. gave and/or received grooming without any interruption. Thus, a grooming bout for individual i started whenever it started to groom another individual or started receiving grooming from another individual, given that individual i was not already engaging in grooming before. The grooming bout ended, whenever individual i neither received nor gave any grooming anymore.

Scanning and movement behavior was also executed in bouts, where a bout was defined as the duration of the time interval an individual continuously engaged in scanning or movement, respectively, without any interruption. The exact durations of all scanning and movement bouts were recorded per individual. The percentage of time an individual was employing scanning or movement behavior was then calculated as the total duration of an individual’s scanning or movement bouts divided by the total recording time, i.e. one YEAR. The movement bout distance was defined as the distance (in meters) an individual moved during one movement bout. Per individual, all movement bout distances were recorded.

To assess the average dyadic proximity score, i.e. the average rate of being located in each other’s proximity, we scored for each individual which other group members were found within close proximity (1 m) at the time of sampling using the one-zero sampling technique. Thus per dyad, possible scores were 1 (in proximity) or 0 (not in proximity) per sample. Note, that the dyadic proximity score is by definition a symmetric measure. Per individual this translates into possible scores between 0 (no other group member was in proximity) and 19 (all other group members were in proximity).

### 12. Initialization

At the initialization of each simulation run, the x-coordinates and the y-coordinates of the 20 individuals were drawn randomly from a predefined circular sphere with an arbitrary diameter of 50 m. Each individual’s initial heading was set to a random orientation between 1° and 360° and the initial view angle was set to 120° for each individual. The dyadic affiliative attitudes (LIKE attitudes) from ego to all other group members were initialized at 0.0. Each individual’s level of arousal was set to the default arousal level (0.09) and the level of anxiety and satisfaction was set to 0.0. Initially, the limit values of arousal, anxiety and satisfaction (i.e. the levels that arousal, anxiety or satisfaction will approach over time) were set to the same values as the initial levels of arousal (0.09), anxiety (0.0) and satisfaction (0.0). However, individuals that perceived any higher-ranking group member, i.e. any group member towards whom they directed a FEAR attitude >0, within 5 m (PERS_DIST), adjusted their limit values for arousal and anxiety, causing arousal and anxiety to increase over time (see *Submodels Arousal* and *Anxiety* for more details). Furthermore, the initial schedule time for each individual was drawn randomly from a normal distribution with a mean of 1 minute and a standard deviation of 0.05 minutes. Table S1 summarizes the initial settings.

### 13. Submodels

This section describes the main procedures of the EMO-model in more detail and covers the implementation of the emotional state (arousal, anxiety and satisfaction), the partner-specific emotional attitudes (LIKE and FEAR), action selection, perception and signaling, scanning, movement, grouping, resting, grooming and counter-attack and escalated fight.

#### Arousal

In our model, an individual’s arousal level, i.e. its responsiveness or activity, increases in response to receiving, executing or observing aggression or when in proximity of a dominant individual. On the other hand, arousal may decrease in response to receiving submissive or affiliative behavior and executing affiliative behavior (see Figure 2). To implement arousal in our model, we used an array of empirical heart rate and scratching rate data from different social contexts (e.g. baseline, post-conflict, grooming). This parameterization procedure is described in Text S1. In our model arousal level was scaled between 0 (inactive) and 1 (maximum stimulation).

We distinguished between point behaviors, which affect the arousal level instantly, and duration behaviors or social contexts, for which the effect on arousal depends on the duration of a behavior or context. Point behaviors that increase arousal in our model are ‘Escalated fight observed’, ‘Attack received’, ‘Attack given’ and ‘Aggressive signal received’. Point behaviors that decrease arousal levels are ‘Submissive signal received’ and ‘Affiliative signal received’. The extent of the arousal change depends on the impact of the stimulus (Table S5). For instance, receiving an aggressive signal or observing an escalated fight is less arousing than receiving an attack (i.e. contact aggression). Upon several aggressive events arousal gets increased per event and may build up quickly up to the maximum possible arousal level of 1.0 (MAX_AR_LIMIT). Submissive and affiliative signals may decrease increased arousal levels towards the baseline level of 0.09 (DEF_AR_LIMIT), but not below (see Table S5).

Duration behaviors that decrease arousal levels in our model are ‘Grooming received’ and ‘Grooming given’, while ‘Perceived proximity of a dominant individual’ is a social context that increases arousal levels. For the duration of such behaviors or contexts, arousal changes with a constant rate towards a context-specific maximum or minimum level, i.e. a limit value that is approached over time. Whenever such a behavior or context is ended, within a few minutes arousal quickly decreases or increases again back to the baseline level, with the default rate of 0.02/min. Thus, the rate of continuous arousal change and the level that is approached over time depends on the current social context (Table S5). For instance, during ‘Grooming received’ arousal decreases faster and towards a lower level compared to the default arousal decrease over time.

Thus, arousal levels change in response to social interactions, which, in turn will affect the individual’s state of responsiveness and activity. In this way, arousal regulates social behavior on a short-term timescale. In our model, higher arousal was implemented to result in an increased probability of performing active behaviors (any behavior except resting) (see *Submodel Action Selection*) and in an increased probability to employ social vigilance, i.e. scanning behavior (see *Submodel Scanning*).

#### Anxiety

In our model, anxiety level, i.e. an individual’s general ‘fearfulness’ in response to negative stimuli within the current social environment, was scaled between 0 (not anxious, DEF_ANXIETY) and 1 (anxious, MAX_ANXIETY). The level of anxiety in our model gets increased instantaneously in response to negative point behaviors, namely ‘Receiving or Giving an attack’, ‘Receiving an aggressive signal’, ‘Losing an (escalated) fight’ or ‘Observing an escalated fight nearby’. The level of anxiety gets decreased instantaneously after positive point behaviors, namely upon ‘Receiving a submissive or affiliative signal’ or after ‘Winning an (escalated) fight’. After an anxiety increase (e.g. due to received aggression) anxiety decreases again over time with a default linear rate of 0.002/min (DEF_ANX_DEC) towards baseline levels (DEF_ANXIETY). Thus, anxiety is assumed to decrease slower than arousal, i.e. within a few hours. Whenever ego engages in grooming, anxiety levels decrease with faster rates, namely 0.01/min for the groomer (GG_ANX_DEC) and 0.02/min (GR_ANX_DEC) for the groomed individual. Whenever grooming is ended, anxiety decreases with the default rate again. Thus, the extent and the rate of anxiety change depend on the current social context. The exact values and rates of anxiety increase and decrease in our model were chosen arbitrarily and are summarized in Table S5.

The level of anxiety in turn affects ego’s valuation of its own position and potential risk within the current social environment. Higher anxiety levels result in increased probabilities to execute affiliation and submission. Furthermore, aggression probability is generally decreased, but is increased towards much lower-ranking partners, resulting in aggression to be more conservative and risk-avoiding (see *Submodel Action Selection* for details).

#### Satisfaction

In our model, satisfaction level, i.e. an individual’s general ‘contentedness’ in response to positive stimuli within the current social environment, was scaled between 0 (not satisfied, DEF_SATISFACTION) and 1 (satisfied, MAX_SATISFACTION). Whenever ego engages in grooming, satisfaction levels increase with linear rates, namely 0.05/min for the groomer (GG_SAT_INC) and 0.1/min (GR_SAT_INC) for the groomed individual. Whenever grooming had stopped, satisfaction decreases again to baseline level (DEF_SATISFACTION) with a default linear decrease rate of 0.02/min (DEF_SAT_DEC), i.e. within one hour. Thus, the extent and the rate of satisfaction change depend on the current social context. The rates of satisfaction increase and decrease in our model were chosen arbitrarily and are summarized in Table S5.

The level of satisfaction in turn affects ego’s valuation of its own affiliative motivation and need within the current social environment. In our model, higher satisfaction levels result in decreased probabilities of (further) affiliation (see *Submodel Action Selection* for details).

#### FEAR attitudes

In our model, individuals assign a partner-specific FEAR attitude to each group member. The FEAR attitude resembles the difference in dominance strength between the individual and the respective group member. Although FEAR attitudes are fixed over the course of our simulation and are, thus, not affected by social interactions, they do affect the individual’s valuation of its potential aggression risk related to the respective group member. A high FEAR attitude results in decreased probabilities of aggression (i.e. attack, aggressive signal) and increased probabilities of submission (i.e. leaving, submissive signal, avoidance) towards the respective group member (see *Submodel Action Selection* for details).

FEAR attitudes are calculated from the (known or perceivable) rank distance towards group members as FEAR_ij_ = myDOM_j_-myDOM_i_, where i is the owner of the FEAR attitude, j is the subject the FEAR attitude is directed to and myDOM is the dominance strength of the respective individual. Thus, FEAR attitudes may have values ranging from −0.95 (directed from highest to lowest-ranking individual) to +0.95 (directed from lowest to highest-ranking individual). In other words, while positive FEAR attitudes (FEAR>0.0) are directed towards higher-ranking individuals and represent actual FEAR, negative FEAR attitudes (FEAR<0.0) are directed towards lower-ranking individuals and represent certain superiority. FEAR attitudes are, thus, directional and not symmetric.

#### LIKE attitudes

In the EMO-model, LIKE attitudes are implemented as partner-specific satisfaction levels, as perceived by ego. LIKE attitudes are thus not necessarily symmetric between two individuals. LIKE attitudes are dynamic in our model. Upon receiving grooming, the level of an individual’s LIKE attitude assigned to the groomer may increase quickly. Thus, LIKE_ij_ reflects individual i’s valuation of individual j, concerning received affiliation. Several satisfaction responses, associated with several episodes of grooming, are integrated over a certain timeframe. LIKE attitudes slowly decrease over time (within days or weeks), depending on the current level of the LIKE attitude.

Ego has a higher probability to direct affiliative behaviors (i.e. grooming, affiliative signals, approach) towards individuals that it assigned a high LIKE attitude to (LIKEd individuals) than towards group members with low LIKE attitudes (less-LIKEd individuals) (see *Submodel: Action Selection* for details). In this way, LIKE attitudes may regulate the development and maintenance of partner-specific affiliative relations. In our model we implemented different settings of the degree to which LIKEd group members are preferred as affiliation partners over less-LIKEd ones (see *Submodel: Action Selection* for details).

LIKE attitudes may have values ranging from 0.0 (neutrally valued affiliation partner) to +1.0 (highly valued affiliation partner). The increase of LIKE_ij_ depends on individual i’s current increase in satisfaction in response to grooming received exclusively from individual j, described by the partner-specific variable PARTNER_SAT_ij_. When receiving grooming from individual j PARTNER_SAT_ij_ increases with the same rate as the general satisfaction level is increasing (GR_SAT_INC). When the specific partner stopped grooming, PARTNER_SAT_ij_ decreases with the same rate as the general satisfaction level is decreasing (DEF_SAT_DEC). Thus, PARTNER_SAT_ij_ is by definition lower than or equal to the individual’s general satisfaction level (mySATISFACTION_i_). Whenever an individual is groomed by several partners simultaneously, the full increase in satisfaction level in response to this grooming is assigned to each groomer.

Partner-specific LIKE attitudes are then used to integrate earlier affiliation received from a partner, i.e. the changing level of PARTNER_SAT_ij_ over time. In contrast to PARTNER_SAT, LIKE attitudes decrease slowly over time. In this way the emotional response to recent affiliation is "remembered" for a while and updated upon renewed affiliation.

The LIKE attitudes are updated as follows:
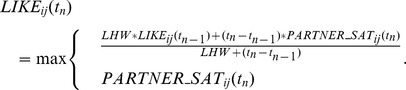
Here, t_n_ is the current time, t_n-1_ is the time of the last update and (t_n_–t_n−1_) is the time since the last update (in MINUTES). LIKE_ij_(t_n_) is the updated value of the LIKE attitude assigned from individual i to j and LIKE_ij_(t_n−1_) is the former level of LIKE to be updated. LHW (LIKE-HISTORY WEIGHT) is a fixed parameter, which describes to which degree the updated LIKE attitude depends on earlier (emotional responses to) affiliation history as opposed to recent (emotional responses to) affiliation. PARTNER_SAT_ij_(t_n_) is the current partner-specific satisfaction level (see above).

Thus, whenever the current level of PARTNER_SAT_ij_(t_n_) exceeds that of LIKE_ij_(t_n_), LIKE_ij_ is instantaneously updated and increased to the same level. Else, the updated level of LIKE_ij_(t_n_) is a weighted combination of the former level of LIKE_ij_(t_n−1_) and the current level of PARTNER_SAT_ij_(t_n_), where the former LIKE attitude is weighted stronger than the current PARTNER_SAT. This was done by setting LHW arbitrarily to 720 MINUTES (1 DAY), while (t_n_–t_n−1_) is usually only a few MINUTES. In this way, short-term fluctuations of PARTNER_SAT will only have minor impacts on LIKE, while a certain regularity and quantity of affiliative interactions is necessary to maintain a high LIKE attitude on a more long-term basis.

When no affiliative behavior was received recently from the respective partner j PARTNER_SAT_ij_ = 0 and LIKE_ij_ will slowly decrease over time. Then LHW can be seen as the half-life of LIKE attitudes, which determines the time it takes before the LIKE attitude decreases to half its value. For instance, when individual i has developed a LIKE attitude of LIKE = 0.8 towards individual j and is afterwards receiving no further affiliation from this individual, LIKE_ij_ will decrease to 0.4 within 1 DAY (LHW = 720 MINUTES = 1 DAY). In sum, current affiliation received from a partner may quickly increase LIKE and/or maintain a high level of LIKE, while the lack of current affiliation will result in a slowly decreasing LIKE attitude.

#### Action selection

In our model, activated individuals may select one of various possible actions. These actions may be directed to other individuals or may involve resting or random movement within the group.

The probability to execute a specific behavior towards another group member depends on a) the distance of the individual to ego, b) ego’s emotional state (arousal, anxiety and satisfaction), c) ego’s FEAR and LIKE attitudes directed to the individual and d) the parameter setting of LIKE-PARTNER SELECTIVITY (LPS), i.e. the degree to which LIKE attitudes are important during partner selection. The emotional state facilitates behavior that is appropriate to the individual’s position and situation within the social group in general, while emotional attitudes facilitate behavior that is appropriate towards specific group members.

First, the 10 (or less) potential interaction partners are determined. Then the possible behaviors towards each of these individuals are determined dependent on their distance to ego. Finally, the probabilities for the possible behaviors towards each potential interaction partner are calculated. According to these probabilities, one of the possible behavior-partner combinations is randomly selected and executed. The details on the exact calculation of the probabilities for affiliation, aggression, submission and avoidance are described in Text S2 and summarized hereafter.

**Figure 3:**
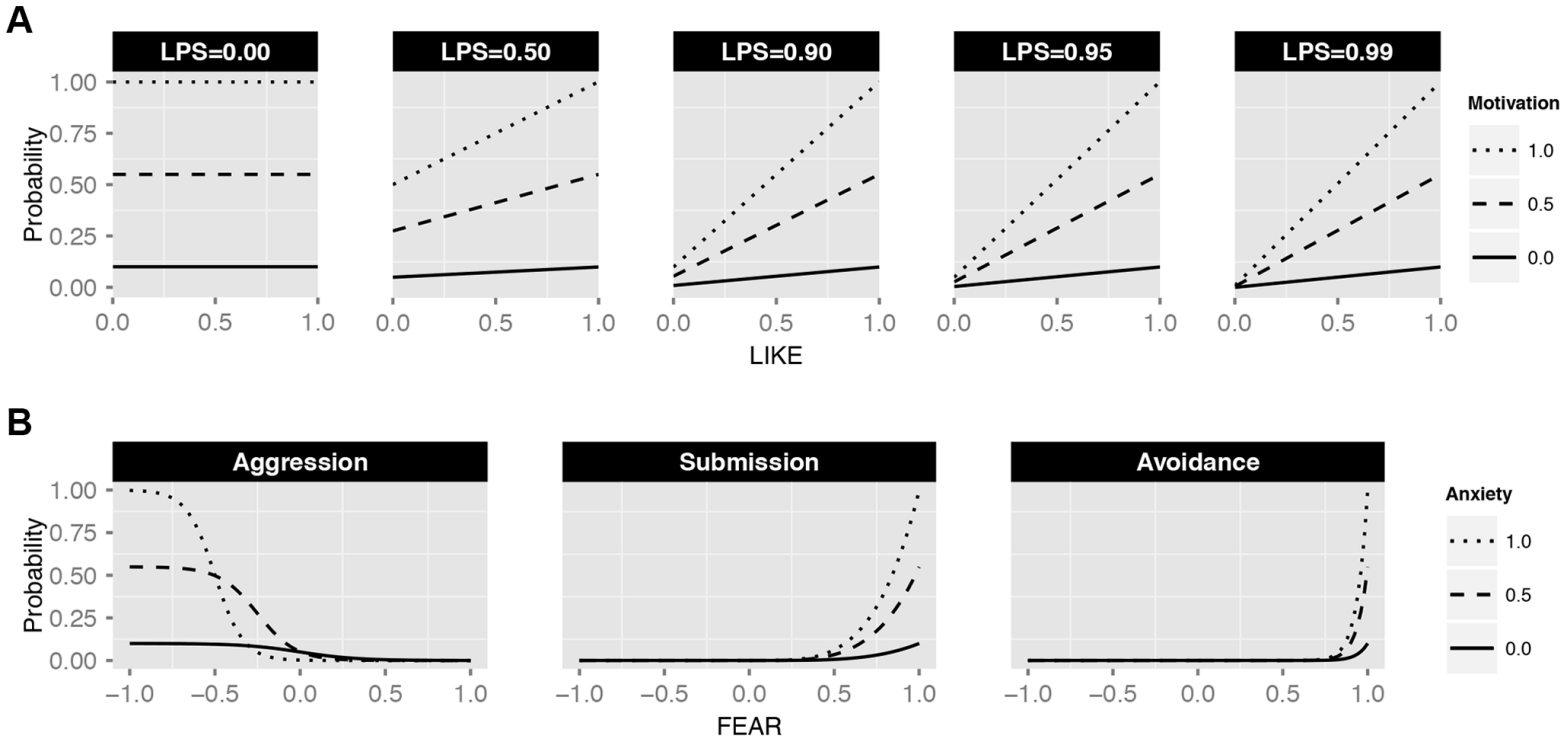
Behavioral probability functions. The upper part of this figure (**A**) shows the general probability for affiliative behavior as a function of the LIKE attitude of ego towards the potential partner (x-axis), depending on the level of an individual’s intrinsic motivation to perform affiliative behavior (dotted line: high motivation, dashed line: intermediate motivation, solid line: no intrinsic motivation) and on the setting of LPS (see panels). The internal motivation is calculated based on ego’s level of anxiety and satisfaction (see text and equation for myAFF_MOT). Higher LPS results in lower affiliation probabilities for potential partners towards whom ego assigns a low LIKE attitude. Thus, with higher LPS ego becomes more selective and prefers high-LIKE partners relatively more than low-LIKE partners. The lower part of this figure (**B**) shows the probability for agonistic behavior as a function of the FEAR attitude of ego towards the potential partner (x-axis), depending on the level of ego’s anxiety (dotted line: high anxiety, dashed line: intermediate anxiety, solid line: no anxiety). The panel shows the specific behavioral probabilities for aggression, submission and avoidance.

Ego’s probability to direct affiliation, i.e. grooming, affiliative signaling and approaching, towards individual j increases with increased LIKE_ij_ (given that LPS>0) and with increased intrinsic affiliation motivation of ego (compare solid, dashed and dotted lines in Figure 3A). Ego’s intrinsic affiliation motivation increases when satisfaction is low or anxiety is high. LPS is the degree to which LIKE attitudes are of importance for the affiliation probability. At LPS = 0, the level of LIKE_ij_ has no effect on the affiliation probability (see first panel of Figure 3A). The higher LPS, the more the affiliation probability depends on LIKE (see the steeper slopes of the lines at increased LPS in Figure 3A).

Ego’s probability to direct aggression, i.e. attacking and aggressive signaling, towards individual j decreases with increased FEAR_ij_ (see monotonously decreasing curves in the first panel of Figure 3B). Moreover, increased anxiety results in the aggression probabilty to be more conservative or risk avoiding. In other words, at increased anxiety levels, the sigmoid curve is steeper and shifted more to the left (compare solid, dashed and dotted lines in the first panel of Figure 3B), resulting in lower aggression probabilities towards high- and similar-ranking individuals, and in higher aggression probabilities towards very low-ranking opponents.

Ego’s probability to direct submission, i.e. leaving and submissive signaling, towards individual j increases with increased FEAR_ij_ and with increased anxiety (compare solid, dashed and dotted lines in the second panel of Figure 3B). Ego’s probability to direct avoidance towards individual i is very similar to the submission probability. However, avoidance is only directed towards individuals that are much higher in rank. Hence, the probability only increases when FEAR is very high (compare second and third panel of Figure 3B).

#### Perception and signaling

Individuals in our model can individually recognize other group members within a maximum perceivable distance of 50 m (MAX_DIST) and within the currently employed view angle. The view angle is by default 120° (VIEW_ANGLE) or else 360° (MAX_ANGLE) when ego is scanning. Group members within 1 m (INTERACT_DIST) are always perceived by ego, even when these individuals are located outside ego’s currently employed view angle.

Moreover, model entities can judge whether at least three other group members are present within 20 m (NEAR_DIST) and within the currently employed view angle. Furthermore, individuals in our model are capable to judge whether their distance to the furthest group member exceeds 100 m (FAR_DIST). The two latter criteria are used by ego to decide whether grouping behavior should be executed.

When an attack escalates into a fight (i.e. is followed by a counter-attack), other group members within 5 m (PERS_DIST) are able to perceive this behavior, even if this event took place outside of their currently employed view angle, since we assume that escalated fights are accompanied by a lot of noise.

Individuals can also perceive signals, which were (‘intentionally’) directed at them from others within 5 m (PERS_DIST). In our model, individuals only direct signals towards others that are also oriented towards them and are thus able to receive the signal. Thus, a sender of a signal is always located within the signal receiver’s currently employed view angle.

Finally, whenever individuals interact with another individual, the visual orientation of the actor and the receiver are set towards each other. Similarly, whenever ego observes an escalated fight nearby, its visual orientation is set towards the salient stimulus, i.e. the counter-attacking individual. If ego (or one of the interacting individuals) was scanning, scanning behavior is stopped whenever attention is attracted by a salient stimulus, i.e. an interaction partner or a counter-attacking individual.

Parameter choices for VIEW_ANGLE, MAX_ANGLE, FAR_DIST, MAX_DIST, NEAR_DIST and PERS_DIST (see Table S2) were adapted from earlier ABM on primate social behavior [49], [61], [66], [67], [79].

#### Scanning

When employing scanning behavior, an individual is turning its head right and left, thus expanding its view angle to 360° (MAX_ANGLE) instead of the default view angle of 120° (VIEW_ANGLE).

Whenever a model individual executes movement or an interaction, we assume that its attention is focused on the movement or the interaction partner. Therefore, individuals in our model may not perform scanning behavior simultaneously with a movement or an interaction. Whenever a movement bout or a social interaction has ended, i.e. whenever ego selected resting, ego may decide to execute scanning behavior. Thus scanning may optionally accompany resting behavior. The probability to engage in scanning behavior increases with increased arousal (see Text S2 for more details). Moreover, in this model, we implemented scanning as duration behavior. Once ego selected scanning, it stays “in scanning mode” until its next activation.

#### Movement

Concerning movement behavior, individuals in our model may either move towards (approaching, grouping) or from (fleeing, leaving and avoiding) other group members or they may execute random movement within the group. Individuals in our model move with a constant speed of 0.6 m/s, which is reasonable for macaques [93].

In contrast to earlier models [66], [67], movement behavior in the EMO-model takes time and is implemented as movement bouts. After starting a movement bout ego is activated each 3 SECONDS to execute the last movement step and to decide whether movement is to be continued (see Text S3 for more technical details). After ending a movement bout ego always performs a proximity update. Ego checks whether any individuals towards which it assigned a positive FEAR attitude (i.e. higher-ranking group members) are now (or still) perceived within 5 m (PERS_DIST), as this proximity of a potential aggressor has consequences for the level that ego’s arousal will approach over time (myAROUSAL_LIMIT). Additionally, also other individuals who assigned a positive FEAR attitude towards ego are updated on ego’s new spatial location, which includes potential updating of their myAROUSAL_LIMIT, if necessary.

Note, that in the EMO-model, fleeing, leaving and avoidance behavior are executed in the same way, i.e. ego moves away from a specific group member. What differs between these behaviors is the context in which they are executed. Leaving defines spontaneous movement away from another individual that is in close proximity, i.e. a potential aggressor. Avoiding defines movement away from another individual, i.e. a potential aggressor that is not (yet) in close proximity. Fleeing defines movement away from an opponent after an attack or an escalated fight. This allows us to implement different probabilities, depending on the specific social context.

#### Grouping

Before selecting a social behavior, model entities always check whether grouping should be executed. Grouping will be selected if less than three (MIN_OTHERS) group members are located within 20 m (GROUP_DIST) and 360° (MAX_ANGLE) or whenever any group member is further away from ego than 100 m (FAR_DIST). When grouping is to be performed, ego simply approaches any randomly selected group member. Grouping was implemented in the model to prevent the group from splitting up into subgroups.

#### Resting

In our model, resting behavior is implemented as a duration behavior, which is executed in bouts. When starting a resting bout, ego’s next activation is scheduled several minutes later to choose its new behavior. Note, that the actual duration of the resting bout may be longer than this schedule time whenever ego selected to continue resting, and may be shorter, whenever ego got activated before it was scheduled (due to receiving an attack or a signal or due to observing an escalated fight nearby).

#### Grooming

In our model, grooming behavior is implemented as a duration behavior, which is executed in bouts. When starting a grooming bout, ego’s next activation is scheduled several minutes later to choose its new behavior. Note, that the actual duration of the grooming bout may be longer than this schedule time whenever ego selected to continue grooming the same individual subsequently. On the other hand the actual duration of the grooming bout may be shorter, whenever ego got activated before it was scheduled, due to receiving an attack or a signal or due to observing an escalated fight nearby. The average schedule time when grooming was set to 7.5±0.375 min (mean ± SD), which resulted in average grooming bout durations of around 6.6 min, which was reasonable for macaques [94]. See *Results: Model validation* for more details.

In our model, receiving grooming was implemented to affect the arousal level and the emotional state of the groomed individual. Moreover, model individuals may receive grooming by two or more groomers simultaneously. For such a scenario, we assumed that the number of simultaneous groomers did not affect the impact of grooming on the arousal level and the emotional state of the groomed individual. In other words, whether ego receives a certain amount of grooming by a single or various groomers simultaneously this results in the same update for ego’s arousal and emotional state. Grooming also affects the LIKE attitudes associated with other individuals. Here, we assumed that ego’s LIKE attitude towards each of several simultaneous groomers is increased with an equal rate as its LIKE towards a single groomer.

#### Counter-attack and escalated fight

Upon receiving an attack the respective model individual is immediately activated to respond with either fleeing or a counter-attack. Moreover, this initial attack immediately increases the anxiety level of the attacked individual. The probability to respond with a counter-attack is calculated using the general aggression probability, which is also used to calculate the probability of a spontaneous attack (see Text S3). Thus, due to ego’s anxiety increase in response to the initial attack, ego’s probability to execute a counter-attack is calculated more conservatively and risk-avoiding compared to ego’s probability to attack the opponent without receiving an attack beforehand (see Text S3). In other words, an attack from a much lower-ranking opponent increases the probability to counter-attack, while an attack from similar- or higher-ranking opponents decreases the probability to counter-attack.

When a counter-attack was selected in response to an attack, we call this an escalated fight. The winner and loser of such an escalated fight are determined randomly according to the individuals’ win chance *w_ij_* (cf. [66]):
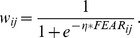



When no counter-attack was selected in response to an attack, the attacked individual is defined as the loser and the attacker as the winner of this aggressive interaction. After an attack or an escalated fight, the loser flees from the winner, while the winner is scheduled anew shortly after.

Whenever an escalated fight takes place, individuals nearby get activated, their arousal level gets increased and their attention is focused at the escalated fight. Moreover, these individuals are activated shortly after to enable an appropriate response to the event. Note that besides avoiding the scene, this may also allow for reactions such as third-party affiliation, coalitionary support or contagion of aggression. However, this was not further studied in this paper.

### 14. Simulation Experiments

In this paper we examined different settings for the parameter LIKE-PARTNER SELECTIVITY (LPS), i.e. the degree to which individuals prefer to affiliate with group members that they assign high LIKE attitudes to. LPS was set to 0.0, 0.5, 0.9, 0.95 or 0.99. LPS = 0.0 resembles a special null model setting. Here, individuals have no preference to select specific affiliation partners concerning LIKE attitudes whatsoever. In other words, individuals do not use LIKE attitudes during affiliative partner selection. Therefore, the null model setting serves as a control setting to assess the effect of the presence of any affiliative partner preference based on emotional bookkeeping.

For each setting of LPS, 10 independent simulations were run, resulting in a total of 50 independent simulation runs.

### 15. Statistical Analysis

We first explain how specific summarized measures were calculated from the recorded data, e.g. how data on dyad level were transformed to obtain measures on individual or (sub-)group level. We continue with the statistics that were used to compare the properties of different subgroups or individual categories. Finally, we explain how we calculated specific group properties, i.e. up/down index, reciprocity and Shannon index, which allowed for quantitative comparison of our model to empirical data. All statistical analyses were performed in R 2.15.2 [95].

Individual proximity scores, strength of LIKE attitudes and behavioral rates were calculated as the sum of all dyadic proximity scores, LIKE attitudes or behavioral rates that an individual directed to others. Group means of behavioral rates were calculated as the mean of all individual behavioral rates. To calculate the mean proximity score, strength of LIKE attitude, emotional states and behavioral rates per rank category, we divided the 20 group members into 10 lower-ranking (subordinates) and 10 higher-ranking (dominants) individuals. We then averaged the individual proximity scores of subordinates or dominants, respectively. Similarly, we averaged the strength of dyadic LIKE attitudes that subordinates or dominants assigned to others, as well as the emotional states and the behavioral rates per subgroup. To calculate the average proximity scores, strength of LIKE attitudes and behavioral rates per rank-distance category, we divided all dyads into two similar-sized groups. Dyads, for which the absolute difference in dominance strength was less than 0.35 were defined as similar-ranking dyads (N = 99 for symmetric measures and N = 198 for directed measures). Dyads, for which the difference in dominance strength was more than or equal to 0.35 were defined as distant-ranking dyads (N = 91 for symmetric measures and N = 182 for directed measures). We then averaged the respective dyadic proximity scores, strength of LIKE attitudes and behavioral rates per subgroup.

To assess the effect of rank categories (subordinates and dominants) or rank-distance categories (similar- and distant-ranking dyads) on proximity scores, strength of LIKE attitudes, emotional states and behavioral rates, we compared the mean values per category over all simulation runs. Data were analyzed using paired t tests with the significance level set at 0.05. For instance the mean grooming rate of subordinates in run i was paired with the mean grooming rate of dominants in run i.

To compare our model to empirical data, we calculated the individual up/down index according to De Waal & Luttrell and Castles et. al [85], [96] as follows:
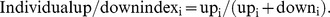



Here, up_i_ is individual i’s rate of a behavior directed to higher-ranking group members, divided by the number of group members that are higher in rank than individual i, while down_i_ is individual i’s rate of a behavior directed to lower-ranking group members, divided by the number of group members that are lower in rank than individual i. This individual up/down index is calculated per individual, except for the lowest- and the highest-ranking group member, and then averaged over those individuals.

To assess the reciprocity of behaviors and LIKE attitudes at the group level we calculated the Kendall’s tau row-wise matrix correlation between the dyadic interaction matrix (or the LIKE matrix) and its transposed [97], [98] using the R software package DyaDA [99].

To assess how evenly individuals distributed their grooming among all potential partners, we calculated the Shannon index (H). This diversity measure has frequently been used in earlier primate research [96], [100]. H of individual i was calculated as:
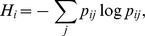
where i is the actor, j are all potential receivers and p_ij_ is the relative proportion of grooming given by the actor i to the jth receiver. We calculated H using the dyadic grooming rates averaged over one YEAR. To compensate for group size, an evenness index was applied to the Shannon index following Buzas & Gibson [101] as:



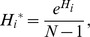
where N is the group size. H* describes whether grooming is directed equally often to all possible partners (H* = 1) or only restricted to one partner (H* approaches 0). H* was calculated per individual and then averaged over the group.

## Results

Below we present the validation of the EMO-model, first describing the behavioral patterns that we aimed to reproduce in our model, and second, describing higher-level patterns that were not explicitly implemented but were emergent properties of our model. Third, we present further patterns that emerged in our model, but of which no empirical data are available yet. These patterns may offer new hypotheses that still have to be validated empirically. Finally, we explain the causation of the patterns in our model and explain the effect of partner selectivity (LPS) on some of these patterns.

### Model Validation

In Table 1, we give an overview of the surveyed empirical data of behavioral patterns, that we aimed to reproduce in our model by tuning a set of model parameters, i.e. the relative action selection probabilities (GG_PROB, AFS_PROB, APP_PROB, ATT_PROB, AGS_PROB, LE_PROB, SS_PROB, AV_PROB, RNDW_PROB and REST_PROB), schedule time when grooming and the chance to end a movement bout (STOP_CHANCE). These parameters were tuned such that the resulting behavioral patterns in our model deviated at most one standard deviation from the mean of the empirical data.

**Table 1:** Model validation and tuned parameters.

Behavioral measure (Unit)	Empirical Mean± SD	Model mean LPS = 0.00	Model mean LPS = 0.50	Model mean LPS = 0.90	Model mean LPS = 0.95	Model mean LPS = 0.99	Species & references for empirical data
Grooming given (min/h)	9.2±8.4	9.9	8.5	7.0	7.0	7.8	M. nemestrina [96], M. arctoides, M. mulatta [85], M. fuscata [108]
Grooming received (min/h)	5.9±3.5	8.1	7.4	6.4	6.5	7.3	M. fascicularis [109]
Time spent grooming GG+GR(% of time)	19.5±11.1	28.3	24.8	20.9	21.0	23.4	M. mulatta [110], [111], M. fuscata [112], [113], M. fascicularis [114], M. sylvanus [115]
Grooming bout duration GG+GR (min)	6.6±5.6	6.3	6.0	5.7	5.7	6.3	M. radiata [94]
Approach rate (1/h)	8.8±8.1	9.7	10.1	9.2	8.1	4.9	M. nemestrina [96], M. assamensis, M. mulatta [87], M. arctoides, M. mulatta [85], M. arctoides [116]
Attack rate (1/h)	0.55±0.87	0.56	0.61	0.68	0.69	0.61	M. nemestrina [96], M. arctoides [85], [116], M. mulatta [85], [111]
Aggressive signal (1/h)	1.46±1.96	0.82	1.01	1.38	1.53	1.83	M. nemestrina [96], M. arctoides [85], [116], M. mulatta [85], [111]
Movement bout distance (m)	6.5±3.5	6.0	5.9	6.1	6.2	6.7	M. mulatta [93]
Time spent scanning (% of time)	4.4±2.3	4.4	5.1	5.7	5.5	4.2	M. fuscata [112], M. fascicularis [109]
Time spent scanning – dominants(% of time)	2.76	2.34	2.72	3.02	2.98	2.34	M. fascicularis [109]
Time spent scanning -subordinates (% of time)	7.05	6.49	7.44	8.34	8.10	6.09	M. fascicularis [109]

Whenever surveyed empirical data sets distinguished between different types of individuals or different conditions, we confined ourselves to those subsets of data that resembled free-living macaques best using the following criteria. Since relatedness is not implemented in our model, data from non-kin were preferred over data from groups with kin relations. Since we did not implement sex differences into our model, data from mixed-sex groups were preferred over data where only one sex was observed. To ensure a consistent way of data recording individual rates of behavior were preferred over dyad-specific data or data that controlled for the number of available partners. Old groups with stabilized relationships were preferred over recently established groups. Groups from vegetated enclosures or enclosures with grass substrate were preferred over groups from non-vegetated enclosures or enclosures with gravel substrate. If data were only presented for subordinates and dominants, we used the average of both groups to estimate a group mean.

Table 1 summarizes the empirical and model data and confirms that the compared measures in our model resemble the empirical data well.

### Higher-level Validation of the Model

We investigated whether our model is able to reproduce higher-level patterns from empirical data.

First, we compared the differences in behavioral patterns between subordinates and dominants (for different settings of LPS) in our model with empirical data of macaques. The following differences between dominants and subordinates that have been shown in macaques were also emerging in our model (see Table 2 for an overview and references of the empirical studies). Subordinates gave less and received more aggression compared to dominants. Subordinates gave more and received less submission than dominants. Subordinates groomed others more often than dominants. Subordinates were more aroused than dominants and engaged more often in scanning and movement behavior. The only emergent pattern that was not fully consistent with empirical data was the amount of grooming received. While some empirical studies have reported that subordinates received less grooming compared to dominants, other studies have found no significant difference between the amount of received grooming of subordinates and dominants. In our model, low LPS resulted in subordinates receiving less grooming than dominants. However, at high LPS (LPS> = 0.9), this pattern was reversed, as subordinates groomed especially other subordinates at these settings. The causation of this pattern in our model will be explained further below.

**Table 2:** Differences between subordinates and dominants.

Behavioral measure	Empirical pattern	Model mean LPS = 0.00	Model mean LPS = 0.50	Model mean LPS = 0.90	Model mean LPS = 0.95	Model mean LPS = 0.99	Species & references for empirical data
**Aggression**							
Aggression given	S<D						M. fascicularis [109], [117], [118]
Attacks given	S<D	S<D***	S<D***	S<D***	S<D***	S<D***	M. fascicularis [117]
Aggressive signal given	S<D	S<D***	S<D***	S<D***	S<D***	S<D***	M. fascicularis [117]
Aggression received	S>D						M. fascicularis [109], [118]
Attacks received		S>D***	S>D***	S>D***	S>D***	S>D***	
Aggressive signal received		S>D***	S>D***	S>D***	S>D***	S>D***	
**Submission**							
Submission given	S>D						M. fascicularis [109], [118]
Leaving given	S>D	S>D***	S>D***	S>D***	S>D***	S>D***	M. fascicularis [117]
Submissive signal given	S>D	S>D***	S>D***	S>D***	S>D***	S>D***	M. fascicularis [117]
Avoidance given	S>D	S>D***	S>D***	S>D***	S>D***	S>D***	M. fascicularis [117]
Submission received	S<D						M. fascicularis [118]
Leaving received		S<D***	S<D***	S<D***	S<D***	S<D***	
Submissive signal received		S<D***	S<D***	S<D***	S<D***	S<D***	
Avoidance received		S<D***	S<D***	S<D***	S<D***	S<D***	
**Affiliation**							
Grooming given	S>D	S>D***	S>D***	S>D***	S>D***	S>D***	M. arctoides, M. fuscata [119], [120]
Grooming received	S<D; NS	S<D***	S<D***	S>D**	S>D***	S>D***	**S<D**: M. fascicularis, M. acrtoides, M. fuscata [118]–[120]; **NS**: M. sylvanus, M. radiata, macaca fuscata [115], [121], [122]
**Emotional state**							
Arousal	S>D	S>D***	S>D***	S>D***	S>D***	S>D***	M. fascicularis [123]
**Other measures**							
Movement	S>D	S>D***	S>D***	S>D***	S>D***	S>D***	M. fascicularis [118]
Scanning	S>D	S>D***	S>D***	S>D***	S>D***	S>D***	M. mulatta [124], [125]

This table summarizes the differences between subordinates (S) and dominants (D) documented in the literature, and compares the findings to the patterns emerging in our model at different settings for LPS. The differences between subordinates and dominants in our model were tested with a paired t-test (N = 10 simulation runs), using significance levels of 0.05 (*), 0.01 (**) and 0.001 (***). The model findings in bold type are contrast to empirical findings.

Second, we compared the differences in behavioral patterns between similar-ranking and distant-ranking dyads in our model (for different settings of LPS) with empirical data of macaques. The following patterns that have been documented for macaques (Table 3) were also emerging in our model. Submissive behaviors were more often directed to distant-ranking than to similar-ranking group members. Affiliative behaviors were more often directed to similar-ranking than to distant-ranking group members. However, note that some studies did not find a significant difference in grooming rates between similar and distant-ranking dyads. Similar-ranking dyads also have been shown to be more often in close proximity than distant-ranked dyads and to have stronger affiliative bonds (as was concluded from the composite sociality index, a measure that involves proximity and grooming rates). Similar-ranking dyads have also been shown to engage more often in aggression than distant-ranked dyads. Our model showed the same pattern considering attacks. However, aggressive signals were only more frequent among similar-ranked dyads than distant ranked dyads, when LPs was high (LPS = 0.99). Else the difference was reversed or not significant in our model. Note, that the difference in frequency of aggressive signals was significant, but only very small at low LPS.

**Table 3:** Differences between similar and distant-ranked dyads.

Behavioral measure	Empirical pattern	Model mean LPS = 0.00	Model mean LPS = 0.50	Model mean LPS = 0.90	Model mean LPS = 0.95	Model mean LPS = 0.99	Species & references for empirical data
**Aggression**							
Aggression	S>D						M. nigra, M. mulatta [126], [127]
Attacks		S>D***	S>D***	S>D***	S>D***	S>D***	
Aggressive signal		S<D***	S<D***	S<D***	**NS**	S>D***	
**Submission**							
Submission	S<D						M. mulatta [128]
Leaving		S<D***	S<D***	S<D***	S<D***	S<D***	
Submissive signal		S<D***	S<D***	S<D***	S<D***	S<D***	
Avoidance		S<D***	S<D***	S<D***	S<D***	S<D***	
**Affiliation**							
Composite sociality index (grooming+proximity)	S>D	S>D***	S>D***	S>D***	S>D***	S>D***	M. mulatta [129]
LIKE		S>D***	S>D***	S>D***	S>D***	S>D***	
PROX	S>D	S>D***	S>D***	S>D***	S>D***	S>D***	M. mulatta [127]
Grooming	S>D; NS	S>D***	S>D***	S>D***	S>D***	S>D***	**S>D**: M. mulatta, M. arctoides, M. fuscata [100], [119], [130]; **NS**: M. arctoides, M. tonkeana [87], [131]
Affiliative signal		S>D***	S>D***	S>D***	S>D***	S>D***	
Approach	S>D	S>D***	S>D***	S>D***	S>D***	S>D***	M. mulatta [127]

This table summarizes the differences between similar (S) and distant-ranked dyads (D) documented in the literature, and compares the findings to the patterns emerging in our model at different settings for LPS (N = 10 simulation runs), using significance levels of 0.05 (*), 0.01 (**) and 0.001 (***). The differences between similar and distant-ranked dyads in our model were tested with a paired t-test. The model findings in bold type are contrast to empirical findings.

Third, we compared some additional group patterns that emerged in our model with empirical data from macaques (Table 4). The individual up/down index [85], [96] of approach was close to 0.5 in our model as well as in empirical data, suggesting that individuals did not prefer to approach especially individuals higher or lower in rank. The individual up/down index of grooming suggested that grooming was slightly more directed up the hierarchy in the empirical data than in our model. The causation of this pattern in our model will be explained further below. However, the mean values of our model were still within one standard deviation of the mean of the empirical data.

**Table 4:** Group patterns.

Behavioral measure	Empirical Mean ± SD	Model mean LPS = 0.00	Model mean LPS = 0.50	Model mean LPS = 0.90	Model mean LPS = 0.95	Model mean LPS = 0.99	Species & references for empirical data
**Individual up/down index**							
Approach	0.44±0.11	0.49	0.45	0.44	0.44	0.45	M. arctoides, M. mulatta, M. nemestrina [85], [96], [116]
Grooming	0.8±0.34	0.62	0.58	0.55	0.54	0.53	M. arctoides [116]
**Reciprocity Kendall tau_rw_**							
Grooming	0.41±0.23 (Range: −0.02–0.74)	−0.10	0.39	0.66	0.73	**0.84**	M. fuscata, M. arctoides, M. fascicularis [119], [120], [132]–[134]
**Shannon index**							
Grooming	0.52±0.19 (Range: 0.30–0.86)	**0.96**	**0.91**	0.80	0.73	0.51	M. fuscata, M. mulatta, M. assamensis, M. nigra, M. thibetana [87], [100], [135], [136]

This table summarizes some additional group patterns documented in the literature, and compares the findings to the patterns emerging in our model at different settings for LPS. The model findings in bold type are contrast to empirical findings.

Regarding the reciprocity of grooming at a group level, as measured by Kendall’s tau_rw_, we found that the range of values found in empirical matrices was quite comparable to our model, except when LPS was very high (LPS = 0.99). Lastly, the range of the Shannon index of grooming was only comparable to the empirical data when LPS was high enough (LPS> = 0.9). Else the Shannon index of grooming suggested that individuals in our model were distributing grooming more equally compared to empirical data, as the Shannon index was higher in our model than in empirical data. Interestingly, in the model the tau_rw_ values and the Shannon index of grooming strongly depended on the LPS setting. This will be analyzed more thoroughly in a sequel paper.

### Model-generated Predictions

We present some additional group-level patterns and differences between dominants and subordinates that emerged in our model. To our knowledge, no empirical data are available yet of these patterns. Therefore, these results (presented in Table 5) may serve as predictions from our model and point out which empirical data are still needed to further validate our model.

**Table 5:** Patterns emergent from our model.

Behavioral measure	Model meanLPS = 0.00	Model meanLPS = 0.50	Model meanLPS = 0.90	Model meanLPS = 0.95	Model meanLPS = 0.99
**Differences between subordinates and dominants**					
Affiliative signal given	Sub>Dom***	Sub>Dom***	Sub>Dom***	Sub>Dom***	Sub>Dom***
Affiliative signal received	Sub>Dom***	Sub>Dom***	Sub>Dom***	Sub>Dom***	Sub>Dom***
Approach given	Sub>Dom***	Sub>Dom***	Sub>Dom***	Sub>Dom***	Sub>Dom***
Approach received	Sub>Dom***	Sub>Dom***	Sub>Dom***	Sub>Dom***	Sub>Dom***
Anxiety	Sub>Dom***	Sub>Dom***	Sub>Dom***	Sub>Dom***	Sub>Dom***
Satisfaction	Sub<Dom**	Sub>Dom*	Sub>Dom***	Sub>Dom***	Sub>Dom***
LIKE given	Sub<Dom***	Sub<Dom***	Sub<Dom***	Sub>Dom***	Sub>Dom***
LIKE received	Sub>Dom***	Sub>Dom***	Sub>Dom***	Sub>Dom***	Sub>Dom***
**Individual up/down index**					
Attack	0.23	0.22	0.21	0.21	0.24
Aggressive signal	0.15	0.15	0.15	0.15	0.18
Affiliative signal	0.48	0.47	0.47	0.48	0.51
**Reciprocity Kendall tau_rw_**					
Approach	0.48	0.68	0.83	0.87	0.90
Affiliative signal	0.35	0.55	0.70	0.76	0.84
LIKE	−0.10	0.43	0.71	0.79	0.90

This table summarizes the emergent patterns from our model (for different settings of LPS), for which empirical data are still needed. The differences between subordinates (Sub) and dominants (Dom) in our model were tested with a paired t-test (N = 10 simulation runs), using significance levels of 0.05 (*), 0.01 (**) and 0.001 (***).

Our model predicted that subordinates have generally higher levels of anxiety compared to dominants. Moreover, subordinates in our model executed and received approach and affiliative signals more frequently than dominants. Furthermore, individuals assigned higher or more frequent LIKE attitudes to subordinates than to dominants. These patterns are similar (and connected) to the pattern of grooming, another affiliative behavior, found in our model (compare Table 2).

For the level of LIKE attitudes that individuals directed to others, and the related level of satisfaction, both of which are affected by received grooming, our model predicted rank-differences that depended on the degree of partner selectivity (LPS). Low LPS resulted in higher LIKE attitudes towards others and higher satisfaction levels for dominants than for subordinates. At high LPS the pattern was reversed and subordinates assigned higher LIKE attitudes to others and had higher satisfaction levels than dominants.

In our model, attacks and aggressive signals were more often directed to lower- than to higher-ranking group members, while affiliative signals were equally often directed to lower- as to higher-ranking group members.

In our model, reciprocity of approaching, affiliative signals and LIKE attitudes strongly depended on the LPS setting, with increased LPS resulting in higher reciprocity. This will be analyzed more thoroughly in a sequel paper.

### Causation of Rank Differences and the Effect of Partner Selectivity

**Figure 4:**
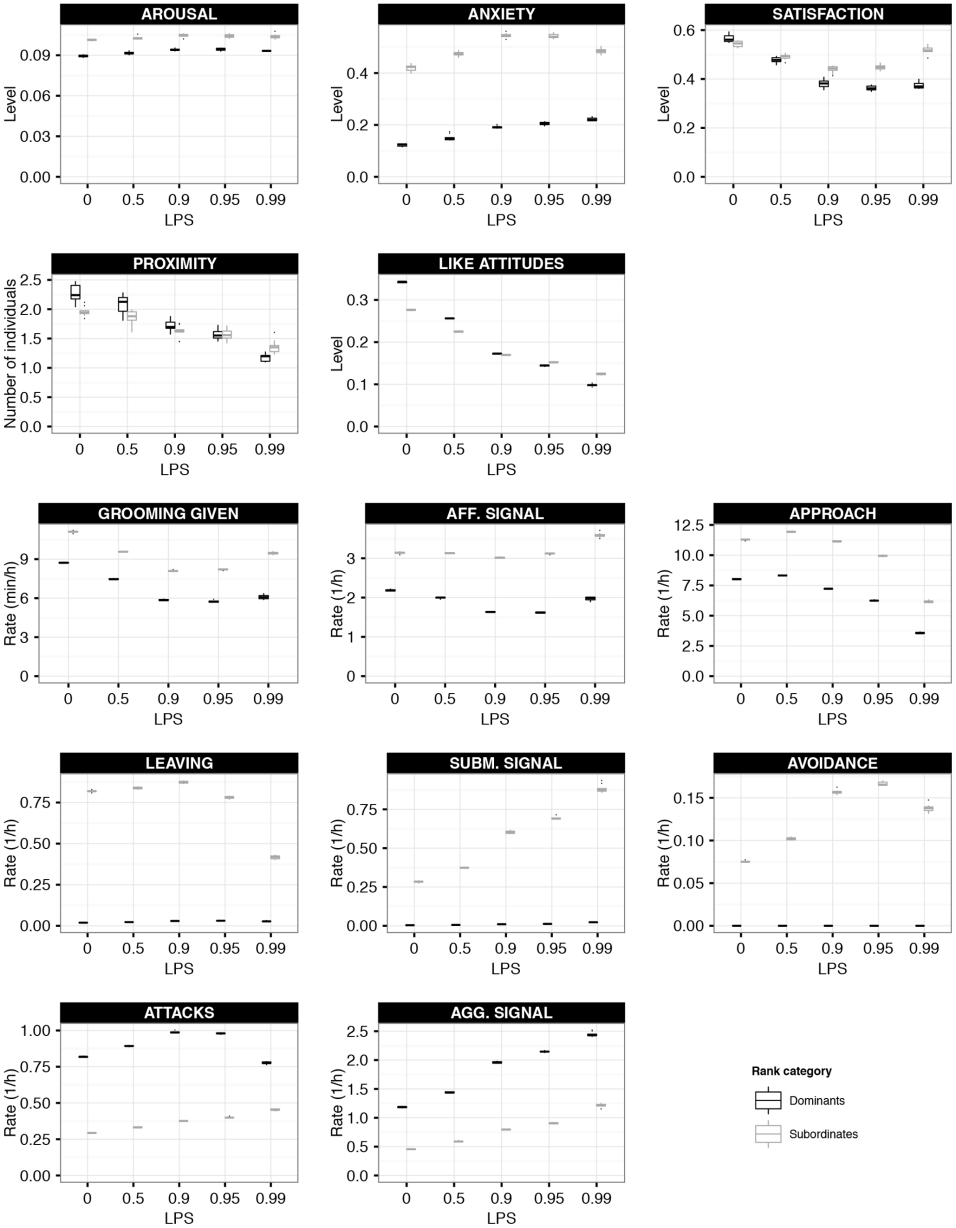
Emotional levels and behavioral rates per rank category. This figure shows the averaged levels of the emotional state and rates of behavior for dominants (black box-plots) and subordinates (grey box-plots) at different settings of selectivity (LPS). Proximity is measured as the average number of individuals in proximity. The LIKE attitudes were measured as the average level of all dyadic LIKE attitudes an (subordinate or dominant) individual directed to other group members. Grooming given is measured in MINUTES per HOUR per individual. Signals, approach, leaving, avoid and attacks are measured in occurrences per hour given per individual. Levels of arousal, anxiety and satisfaction levels were averaged per individual. The box-plots show the results of 10 simulation runs, averaged over 1 YEAR.

Here, we explain the causation of the patterns of the EMO-model concerning differences between dominants and subordinates in their emotional states (level of arousal, anxiety and satisfaction) and behavioral patterns. We explain the patterns for the null model setting (LPS = 0), where LIKE attitudes do not affect the affiliative partner selection of individuals, and assess the effect of increased partner selectivity (LPS). Results are depicted in Figure 4, unless indicated otherwise. Note, that in the EMO-model levels of anxiety (and arousal) get increased upon giving, receiving or perceiving aggression, while they decrease upon receiving submissive signals or when engaging in affiliation. On the other hand, levels of satisfaction increase upon engaging in grooming.

LPS, i.e. partner selectivity, determined the degree to which individuals preferred to selectively affiliate with partners that were assigned a high LIKE attitude. In the null model setting (LPS = 0), where individuals had no preference for specific affiliative partners, interaction patterns were mainly driven by the dominance relations in the group. Individuals avoided mainly distant-ranking group members (Table 3), resulting in higher proximity scores and affiliation rates among similar-ranked than among distant-ranked dyads (Table 3). At LPS>0, affiliation feeds back onto the LIKE attitudes. LIKE attitudes decrease towards partners that do not groom an individual regularly enough (i.e. distant-ranking group members), while they are maintained towards regular groomers (i.e. similar-ranking group members). At increased LPS, this differentiation in LIKE attitudes gets reinforced, as individuals almost exclusively groom regular groomers. This is also reflected in the Shannon index of grooming, which decreases at increased LPS (Table 4).

In our model, subordinates always had higher levels of arousal and anxiety than dominants, independently of the setting of LPS. Lower win chances of subordinates were explicitly implemented in our model. As a result, subordinates received aggression more frequently than dominants. At increased LPS, anxiety levels, and to a smaller extent also arousal levels, were generally increased for both, subordinates and dominants. This was mostly a result of generally increased aggression rates (see also below). Only at high LPS (LPS>0.9), subordinates’ levels of anxiety decreased again due to increased rates of affiliation in these settings (see also below). In the null model setting (LPS = 0), dominants had slightly higher satisfaction levels than subordinates, as they received more grooming than subordinates (see Table 2). At increased LPS, satisfaction levels first decreased (due to more selective affiliation at high LPS), and this decrease was stronger for dominants than for subordinates. At high LPS (LPS>0.9) subordinates’ satisfaction levels increased again. This is explained below.

Proximity indices and the values of LIKE attitudes were always quite similar for dominants and subordinates and they generally decreased at increased LPS due to more selective, i.e. decreased, affiliation. While at low LPS (LPS< = 0.5) dominants had slightly higher proximity scores and directed higher average LIKE attitudes to others than subordinates, this was reversed at maximum LPS (LPS = 0.99). Note, that in the null model setting we measured the (hypothetical) LIKE attitudes resulting from received affiliation. However, individuals did not use this information in this setting. Hence, LIKE attitudes (i.e. emotional bookkeeping) had no effect on any of the behavioral patterns in the null model setting.

At low LPS, affiliative behaviors were slightly more directed up the hierarchy (Table 4) resulting in dominants receiving more affiliation than subordinates (Table 2) and therefore directing higher LIKE attitudes to others and being more often in proximity to others compared to subordinates. At increased LPS, affiliative behaviors were more reciprocated and more restricted to fewer (similar-ranking) individuals (Table 4 and 5). Additionally, subordinates still had higher anxiety levels than dominants. The resulting higher affiliation rates of subordinates were almost exclusively directed at other subordinates at high LPS, causing subordinates to have slightly higher proximity values, to direct slightly higher LIKE attitudes to other (mostly also subordinate) group members and to have higher satisfaction levels than dominants.

As subordinates always had higher anxiety levels than dominants, they always engaged more frequently in affiliative behaviors (grooming, affiliative signals, approach) than dominants, independently of the setting of LPS. At increased LPS, approach rates generally decreased for all individuals due to more selective affiliative partner choice, which also affected whom to approach. Also, grooming and affiliative signals first decreased for all individuals (due to more selective partner choice), but increased again at high LPS (LPS> = 0.9). Only at high LPS, the affiliative partner selectivity was strong enough for individuals to develop and maintain LIKE attitudes towards a few preferred partners that were high enough to increase affiliation rates such that the decrease of affiliation due to selectivity was counteracted. The decrease of affiliation at increased LPS and the increase at still higher LPS was also reflected in the satisfaction levels. Moreover, the more frequent affiliation among subordinates than dominants at high LPS was also reflected in the decrease in anxiety levels for subordinates.

Dominants always engaged more often in aggressive behaviors (attack, aggressive signals) than subordinates, independently of the setting of LPS, due to their higher rank and thus their higher chance of winning. At increased LPS, aggression rates for both, subordinates and dominants, generally increased, except for the dominants at maximum LPS (LPS = 0.99). This was a result of more selective affiliation at increased LPS, which resulted in decreased affiliation rates, increased anxiety levels and individuals being more often in proximity of similar-ranking than distant-ranking individuals. Moreover, the probability to direct aggression towards similar-ranking is usually higher than towards distant-ranking individuals, except when ego itself was very high-ranking. Therefore, at maximum LPS (LPS = 0.99) attack rates decreased again for dominants, as their opponents were now more similar-ranking, which resulted in lower win chances and, thus, lower probabilities to attack.

Submissive behaviors (leaving, submissive signals, avoidance) were almost exclusively executed by subordinates, as these behaviors were implemented as strictly unidirectional behaviors in our model. Additionally, this was a result of subordinates having generally higher levels of anxiety due to their losing of fights and receiving of aggressive signals. At increased LPS, rates of submissive signals and avoidance increased for subordinates due to their increased levels of anxiety. At maximum LPS (LPS = 0.99) avoidance rates decreased again for subordinates due to selective proximity towards similar-ranking individuals. Similarly, rates of leaving decreased at high LPS (LPS>0.9) due to selective proximity towards similar-ranking individuals.

## Discussion

In this paper, we presented the EMO-model, i.e. the first agent-based model that explicitly incorporates the interrelations between primate social behavior, emotional processes and emotional bookkeeping of affiliative relationships. We integrated an array of empirical data and several partial hypotheses (on general macaque behavior, animal emotions, the two-dimensionality of the emotional system and emotional book-keeping), to obtain an explicitly formulated hypothesis, about how emotional processes may regulate primate social behavior and mediate affiliative relationships [15], [17]. We succeeded in producing a representative model that also generated many structural properties of real macaque groups on multiple levels. While general behavioral frequencies in the model were tuned to empirical data, group-level patterns arose as a result from the incorporated underlying processes without being explicitly implemented into the model. This multi-level validity of our model suggests that the implemented causal relations between processes are plausible [81].

### Substantiation of the Model

While developing the EMO-model we aimed to base each emotional and behavioral process implemented in the model on empirical data. In many respects we succeeded in doing so, but not in all. Yet, to be of value to empirical research, simulation models should be well grounded in empirical data.

We were able to use empirical data on macaques to determine an array of parameter settings of the model entities, such as the active (non-sleeping) time of a day, the distance at which individuals may interact physically, the percentage of time spent grooming or scanning, the duration (minutes) of grooming bouts, the movement bout distance (in meters) and the speed with which individuals move. Based on empirical data, we assumed a stable hierarchy. Moreover, we were able to construct an arousal measure and its dynamics from empirical heart rate and scratching data. However, more data, ideally measured simultaneously during a specific social context, would allow a more reliable comparison and transformation between heart rate and scratching rate data. Ample knowledge was acquired about how arousal levels may differentially increase or decrease in response to received, given or observed behaviors, such as aggression, grooming and perceiving a dominant individual in proximity, as well as how arousal may decrease over time accompanied by affiliation or not. Based on empirical data, we assumed that increased arousal level results in increased social vigilance (scanning) and activity. However, more quantitative data in macaques are needed to substantiate this.

For some parts of our model, parameter settings and processes were based on an educated guess or adapted from comparable modeling studies, since we were unable to find relevant empirical studies. This concerned settings for some general parameters, such as the width of the default field of view of macaques, quantitative activation or reaction times in specific contexts, the number of potential interaction partners taken into account during action selection and the distances at which group members may be perceived, recognized, avoided or signaled to. This also concerned the implemented order of decisions in the decision tree during action selection, grouping criteria and the implementation of the win chance. Moreover, no empirical data were available to validate the unidirectionality of avoidance or leaving. However, as submissive signals were found to be unidirectional, this seemed a plausible assumption.

Quantitative data on how arousal levels may differentially increase or decrease in response to particular received, given or observed behaviors are still needed to further substantiate our EMO-model. This concerns the qualitative impact on arousal of observing aggression and receiving submissive or affiliative signals, as well as the quantitative increase or decrease specific to the social behavior and context, how it depends on the intensity and frequency of the behaviors and the question whether there are indeed context-specific minima/maxima of arousal level.

A few behavioral or physiological traits have been defined as immediate and quantitative markers of negative or positive emotional state (anxiety/satisfaction). However, a positive emotional state is often measured rather in terms of the absence or decrease of negative measures, e.g. stress indicators [12], [102] and measures of a negative emotional state, such as heart rate and scratching rate, often primarily reflect arousal instead of valence [12]. Quantitative measures of the emotional state, which can be distinguished from arousal, would allow a more quantitative substantiation of our model concerning the dynamics of anxiety and satisfaction in response to (social) behavior.

Specifically, the importance of anxiety and satisfaction with respect to the affiliation motivation is yet unclear. In our model, anxiety determines affiliation motivation to a stronger degree than satisfaction. This seemed a reasonable assumption, as we assumed that to decrease anxiety (e.g. in response to received aggression) should have a higher priority than to increase satisfaction (in response to the lack of recent grooming). This anxiety-driven grooming in our model leads to extensive grooming among subordinates and much less grooming in dominants.

### Validation of the Model

Our model represents a plausible framework of emotional regulation of social behavior and affiliative relationships in macaques. While we tuned our model to reproduce general behavioral frequencies that match empirical data of macaques, the model also yielded higher-level patterns similar to macaque groups without explicitly implementing them. Higher-level patterns that match the empirical data strengthen the validity of our model, as those patterns were not intended to be reproduced by the model, but emerged from the complex interactions of the model entities.

In total, we have compared an array of 30 emergent model properties, i.e. differences between subordinates and dominants, differences between similar and distant-ranked dyads and some additional group-level properties, to empirical data. Most of the model patterns were consistent with empirical data. Four of the 30 behavioral patterns in our model were only partially validated and depended on the specific parameter setting of LPS (LIKE-PARTNER SELECTIVITY), i.e. the degree of the preference for LIKEd affiliation partners. This concerned differences in aggressive signaling between similar and distant-ranked dyads, rank differences in received grooming, as well as reciprocity and distribution of grooming.

However, inconsistent differences in aggressive signaling between similar and distant-ranked dyads were only very small in these settings and the causality of the unsupported rank differences in received grooming could be explained within our model (see *Results*). The strong dependence of reciprocity and distribution of grooming on the LPS parameter will be analyzed further in a sequel paper.

In sum, most of the emergent behavioral patterns in our model are consistent with empirical data. While we did not achieve a complete validation of all model patterns, we succeeded in developing a model implementation of emotional bookkeeping, which reproduces social behavioral patterns of macaques sufficiently and specifically allows us to understand how these patterns are affected by the degree of LPS.

Moreover, some additional behavioral patterns were not available from empirical data, but are consistent with measures of related phenomena. The individual up/down indices in our model suggest that attacks and aggressive signals are more often directed to lower- than to higher-ranking group members. This seems consistent with empirical data that suggest higher aggression rates for similar-ranking dyads compared to distant-ranking ones. Furthermore, approaching was very symmetric in our model. This seems consistent with the individual up/down index found in empirical data.

One may criticize our choice to ignore sex differences and to use data of various macaque species for the validation of our EMO-model. Sex ratio within a group may affect the group dynamics and we are aware of the fact that different macaque species show considerable behavioral diversity, for instance concerning the steepness of their hierarchy. There has been already a lot of effort to study this theoretically [65], [103], [104]. We consider these topics outside of the scope of the current study, yet they are worth to be addressed in the future. We do not pursue to fit our model to one specific macaque species. Instead, we aimed to produce a qualitative model, inspired by macaques, to understand the emotional regulation of behavior and emotional bookkeeping. Since the general mechanisms underlying the emotional framework are not expected to differ considerably between macaque species or sexes, we deem our approach valid.

### Model-based Predictions

Our model yielded results that may pinpoint gaps in the empirical data and in the theoretical understanding of the underlying mechanisms. All model-based predictions, i.e. rank-differences in anxiety, satisfaction, giving and receiving of approach, affiliative signals, LIKE attitudes, the direction of attacks, aggressive and affiliative signals and the reciprocity of approach, affiliative signals and LIKE attitudes are connected to patterns of grooming and aggression found in our model and are consistent with these dynamics within our model. Yet, empirical data are still needed to verify these patterns.

Interestingly, our model predicted that the reciprocity of LIKE attitudes and affiliative behaviors, i.e. grooming, affiliative signals and approach, was enhanced at increased partner selectivity. This suggests that emotional bookkeeping may affect reciprocal affiliative relationships to a great degree. This will be explored more thoroughly in our model in a sequel paper. LIKE attitudes, i.e. the internal representation of the differential valuation of grooming partners, cannot easily be assessed quantitatively in real animals. Similarly, the degree of affiliative partner selectivity (LPS), and thus its effect on reciprocity of LIKE attitudes, is a theoretical construct that is tied to the concept of LIKE attitudes, which makes it difficult to assess this in real animals.

### Further Directions

As our model presents an initial attempt to integrate emotional processes and primate social behavior, several processes of the emotional dynamics implemented in this model may be subject to refinement or improvement.

For instance, in the current EMO-model LIKE attitudes quickly increase in response to grooming, and slowly decrease, integrating the recent history of LIKE (and thus earlier grooming). However, the updating of LIKE attitudes may also be directly dependent on the current level of the LIKE attitudes. Some researchers recently have reported long-term, but not necessarily short-term, reciprocity in primates [105], [106]. Thus, in established relationships (internally represented by high LIKE attitudes), renewed grooming, or especially the lack of it, may have less impact compared to newly developing relationships (internally represented by low LIKE attitudes). On the other hand, in recent research on chimpanzees, higher oxytocin levels have been found after grooming with bond partners than after grooming with non-bond partners [107]. Thus, in contrast to the first suggestion, grooming with established bond partners (assigned a high LIKE attitude) might be more rewarding than grooming with non-bond partners.

Given that empirical data are not consistent with the rank-dependent patterns of received affiliation in our model, the model may be partially adjusted to represent the empirical data better. We suggest that a possible starting point might be to more carefully study the importance of anxiety and satisfaction with respect to the affiliation motivation. This should be done empirically, but further exploration of our model with adjusted settings also promises to yield valuable insights. In the current EMO-model, anxiety determines affiliation motivation to a stronger degree than satisfaction, i.e. anxiety is weighted nine times higher than satisfaction. Exploring our model with more similar weighting factors for anxiety and satisfaction in the calculation of the affiliation motivation may yield more plausible simulation results.

Moreover, our model may be extended by further mechanisms. For instance, in the current model, the hierarchy and the FEAR attitudes were fixed and assumed to be stable. An earlier model, GrooFi-world [65], implemented a dynamic dominance hierarchy, as well as agonistic and affiliative behavior. Interestingly, this model generated similar emergent patterns, whether the dominance hierarchy was dynamic or stable. Yet, the GrooFi-world model did not implement LIKE attitudes or any emotional regulation of dynamic dominance ranks. In our EMO-model, FEAR attitudes may be implemented in a dynamic way by integrating the anxiety that was caused by aggression received from a specific group member or by fights that were lost from that individual. As FEAR attitudes in turn affect the future agonistic behavior, this is expected to reinforce the differentiation in FEAR attitudes. How dynamic FEAR attitudes may affect the development and maintenance of LIKE attitudes and vice versa, is interesting to study and may offer new hypotheses for the relation between affiliative and agonistic relationships in macaques. For instance, it is still unclear whether agonistic relationships restrict affiliative relationships, or whether the same partner may for example be assigned a high LIKE and a FEAR attitude simultaneously.

A limitation of the current model is that LIKE attitudes are only affected by grooming. A central aspect of the emotional bookkeeping hypothesis and of general research on emotional processes is that different social interactions may be integrated into a common currency. Our model may be extended by the possibility that also agonistic interactions, such as agonistic support, may affect LIKE. This may result in LIKE attitudes (and resulting grooming) that are directed up the hierarchy, a pattern that is still problematic in the current model, and in an exchange of grooming and support.

Nonetheless, the model presented in this paper serves as a starting point to study the emotional regulation of social processes theoretically and may offer a promising framework to study the complex dynamics of social relationships. The model allows the exploration of how affiliative relationships may be developed and maintained. It will be interesting to study the differentiation and structure of affiliative relationships within the group, and how these patterns depend on certain crucial parameters. Lastly, this model allows the exploration of emotional bookkeeping and its consequences for the duration and stability of social bonds. For instance, it has been proposed that emotional bookkeeping, which integrates earlier partner-specific episodes over a long term, may result in long-term but not necessarily short-term reciprocity of affiliative behavior [105], [106]. Moreover, complex behavioral patterns such as reconciliation, redirection, reciprocity, behavioral contagion or coalitions may emerge in our model and may be explained in terms of the underlying emotional regulation.

The EMO-model will be used in future studies to investigate the effect of two key parameters, LHW (LIKE-HISTORY WEIGHT) and LPS (LIKE-PARTNER SELECTIVITY) on affiliation patterns. LHW determines the extent to which earlier, as opposed to recent, affiliative episodes are integrated into the valuation of affiliation partners via LIKE attitudes. LPS determines the degree to which ‘valuable’ affiliative partners, i.e. partners that are assigned high LIKE attitudes, are preferred as affiliation partners. Exploring the EMO-model for different settings of LHW and LPS will give further insights on the plausible timeframe of emotional bookkeeping and the degree of partner selectivity, necessary for the emergence of long-term reciprocal, individual-specific affiliative relationships.

## Conclusion

The EMO-model assumes that patterns in social behavior may result from two emotional dimensions, anxiety and satisfaction, that form an individual’s current emotional state, in combination with individual-specific FEAR and LIKE attitudes stored in the individual’s social memory due to earlier interactions. Modeling two emotional dimensions mimics at least part of the empirical complexity and these particular emotional dimensions represent two fundamentally different and important emotions. By modeling two emotional dimensions, we chose not to search for the simplest rules generating social complexity, but chose to explore realistic rules and their interactive effects on social patterns. The EMO-model generates several emergent patterns at group level that are consistent with empirical data, in particular in macaques, and some new patterns. This suggests that a (more) realistic representation with emotions of different valence guiding social interactions can yield emergent patterns in behavior. This research tool will allow the disentangling of the organizing effects of the two different emotions anxiety-FEAR and satisfaction-LIKE on sociality.

## Supporting Information

Table S1 **Individual-specific state variables of the model entities.**
(DOC)

Table S2 **General model parameters.**
(DOC)

Table S3 **Schedule times depending on performed, received or observed behavior.**
(DOC)

Table S4 **Interaction distances for social behaviors.**
(DOC)

Table S5 **Effect of social behaviors on arousal, anxiety and satisfaction levels.**
(DOC)

Text S1 **Parameterization of arousal.**
(DOC)

Text S2 **General behavioral probabilities.**
(DOC)

Text S3 **Technical details on movement in bouts.**
(DOC)
