# Supplementary material for: The EMO-Model: An Agent-Based Model of Primate Social Behavior Regulated by Two Emotional Dimensions, Anxiety-FEAR and Satisfaction-LIKE
Source: PLoS One. 2014 Feb 4;9(2):e87955. doi: 10.1371/journal.pone.0087955 (PMC3913693; doi:10.1371/journal.pone.0087955)
Supplement: Table S1 — Individual-specific state variables of the model entities. (DOC) [file pone.0087955.s001.doc]

**Table S1: Individual-specific state variables of the model entities.**

| **State Variable Name** | **Description** | **(Initial) Value** | **Possible Range or Values** | **Fixed / Dynamic** |
| --- | --- | --- | --- | --- |
| GENERAL STATE VARIABLES | | | | |
| myTIME | Waiting time until next scheduled activation | 1 ± 0.05 min (mean ± SD) | May range between 0.0 (attack received) and 7.5 ± 0.375 min (rest/groom) (depending on the social context) | Dynamic |
| myDOM | Dominance strength | Between 1/N and 1.0 | Scaled between 1/N (lowest-ranking) and 1.0 (highest-ranking) | Fixed |
| mySCAN_PROB | Probability of employing scanning | Depends on arousal and current behavior | May range between 0.0 and 1.0 (depending on arousal and current behavior) | Dynamic |
| myVIEW_ANGLE | Width of currently employed view angle | 120º | May be either 120º (not scanning) or 360º (scanning) | Dynamic |
| EMOTIONAL STATE VARIABLES | | | | |
| myAROUSAL | Arousal state | 0.09 | May range between 0.0 (inactive) and 1.0 (aroused) | Dynamic |
| mySATISFACTION | Affiliation-related emotional state | 0 | May range between 0.0 (unsatisfied) and 1.0 (satisfied) | Dynamic |
| myANXIETY | Agonism-related emotional state | 0 | May range between 0 (not anxious) and 1 (anxious) | Dynamic |
| myAROUSAL_LIMIT | Arousal level that is approached over time | 0.09 | May be 0.03 (grooming received), 0.04 (grooming given), 0.09 (default), 0.12 (dominant perceived), 1.0 (aggression) | Dynamic |
| myANXIETY_LIMIT | Anxiety level that is approached over time | 0 | May be 0.0 (not anxious) or 1.0 (anxious) | Dynamic |
| mySATISFACTION_LIMIT | Satisfaction level that is approached over time | 0 | May be 0.0 (not satisfied) or 1.0 (satisfied) | Dynamic |
| EMOTIONAL ATTITUDE VARIABLES | | | | |
| FEAR_ij_ | Agonism-related emotional attitude from individual i to j | myDOM_j_ - myDOM_i_ | myDOM_j_ - myDOM_i_ | Fixed |
| LIKE_ij_ | Affiliation-related emotional attitude from individual i to j | 0 | May range between 0 (neutral) and 1 (preferred affiliation partner) | Dynamic |
