# Supplementary material for: The EMO-Model: An Agent-Based Model of Primate Social Behavior Regulated by Two Emotional Dimensions, Anxiety-FEAR and Satisfaction-LIKE
Source: PLoS One. 2014 Feb 4;9(2):e87955. doi: 10.1371/journal.pone.0087955 (PMC3913693; doi:10.1371/journal.pone.0087955)
Supplement: Table S2 — General model parameters. (DOC) [file pone.0087955.s002.doc]

**Table S2: General model parameters.**

| **Parameter** | **Description** | **Value** |
| --- | --- | --- |
| **General parameters** | | |
| N | Number of individuals in the group | 20 |
| D | Grid unit | 1 m |
| FIELD_SIZE | Field size | 300 x 300 m |
| MINUTE | Time step | 1 min |
| HOUR | 1 hour | 60 MINUTES |
| DAY | 1 day | 12 HOURS |
| WEEK | 1 week | 7 DAYS |
| YEAR | 1 year | 50 WEEKS |
| **Sensing parameters** | | |
| VIEW_ANGLE | Default view angle | 120º |
| MAX_ANGLE | View angle when scanning | 360º |
| FAR_DIST | Maximum tolerated distance to furthest group member | 100 m |
| MAX_DIST | Maximum distance to individually recognize group members | 50 m |
| NEAR_DIST | Maximum preferred distance to the group | 20 m |
| PERS_DIST | Maximum distance to perceive signals or escalated fights | 5 m |
| INTERACT_DIST | Maximum distance to physically interact with others | 1 m |
| MIN_OTHERS | Minimum preferred number of conspecifics in NEAR_DIST | 3 |
| **Movement parameters** | | |
| SPEED | Movement speed | 0.6 m/s |
| STOP_CHANCE | Probability of ending the current movement bout | 0.1 |
