# Supplementary material for: The EMO-Model: An Agent-Based Model of Primate Social Behavior Regulated by Two Emotional Dimensions, Anxiety-FEAR and Satisfaction-LIKE
Source: PLoS One. 2014 Feb 4;9(2):e87955. doi: 10.1371/journal.pone.0087955 (PMC3913693; doi:10.1371/journal.pone.0087955)
Supplement: Table S3 — Schedule times depending on performed, received or observed behavior. (DOC) [file pone.0087955.s003.doc]

**Table S3: Schedule times depending on performed, received or observed behavior.**

| **Behavior** | **Schedule time (mean ± SD)** |
| --- | --- |
| ATTACK received | 0.0 sec |
| Escalated FIGHT observed / SIGNAL received | 0.1 ± 0.005 sec |
| Movement (LEAVE, FLEE, AVOID, APPROACH, RANDOM WALK) | 3 sec |
| FIGHT won / ATTACK given / SIGNAL given | 1 ± 0.05 min |
| GROOMING given / REST | 7.5 ± 0.375 min |
