# Supplementary material for: The EMO-Model: An Agent-Based Model of Primate Social Behavior Regulated by Two Emotional Dimensions, Anxiety-FEAR and Satisfaction-LIKE
Source: PLoS One. 2014 Feb 4;9(2):e87955. doi: 10.1371/journal.pone.0087955 (PMC3913693; doi:10.1371/journal.pone.0087955)
Supplement: Table S4 — Interaction distances for social behaviors. (DOC) [file pone.0087955.s004.doc]

**Table S4: Interaction distances for social behaviors.**

| **Interaction category** | **< 1m** | **< 5m** | **> 1m** |
| --- | --- | --- | --- |
| Affiliation | GROOM | AFFILIATIVE SIGNAL | APPROACH (< 50m) |
| Submission | LEAVE | SUBMISSIVE SIGNAL | AVOID (< 5m) |
| Aggression | ATTACK | AGGRESSIVE SIGNAL |  |
