# Supplementary material for: The EMO-Model: An Agent-Based Model of Primate Social Behavior Regulated by Two Emotional Dimensions, Anxiety-FEAR and Satisfaction-LIKE
Source: PLoS One. 2014 Feb 4;9(2):e87955. doi: 10.1371/journal.pone.0087955 (PMC3913693; doi:10.1371/journal.pone.0087955)
Supplement: Table S5 — Effect of social behaviors on arousal, anxiety and satisfaction levels. (DOC) [file pone.0087955.s005.doc]

**Table S5: Effect of social behaviors on arousal, anxiety and satisfaction levels.**

| **Behavior** | **Change of arousal, anxiety or satisfaction level in the model** | **Parameter name** |
| --- | --- | --- |
| **Behaviors affecting arousal level** | | |
| Escalated fight observed | + 0.04 | EFO_AR_INC |
| Aggressive signal received | + 0.04 | ASR_AR_INC |
| Attack given | + 0.04 | AG_AR_INC |
| Attack received | + 0.08 | AR_AR_INC |
| Affiliative signal received | - 0.04 | AS_AR_DEC |
| Submissive signal received | - 0.04 | SS_AR_DEC |
| Default decrease | - 0.02 / min | DEF_AR_DEC |
| Default increase | + 0.02 / min | DEF_AR_INC |
| Proximity of dominant | + 0.02 / min | PD_AR_INC |
| Grooming given | - 0.02 / min | GG_AR_DEC |
| Grooming received | - 0.04 / min | GR_AR_DEC |
| **Behaviors affecting anxiety level** | | |
| Escalated fight observed | + 0.2 | EFO_ANX_INC |
| Aggressive signal received | + 0.2 | ASR_ANX_INC |
| Attack given | + 0.2 | AG_ANX_INC |
| Attack received | + 0.4 | AR_ANX_INC |
| (Escalated) fight lost | + 0.4 | EFL_ANX_INC |
| Affiliative signal received | - 0.2 | ASR_ANX_DEC |
| Submissive signal received | - 0.2 | SSR_ANX_DEC |
| (Escalated) fight won | - 0.4 | EFW_ANX_DEC |
| Default anxiety decrease rate | - 0.002/min | DEF_ANX_DEC |
| Anxiety decrease rate when giving grooming | - 0.01/min | GG_ANX_DEC |
| Anxiety decrease rate when receiving grooming | - 0.02/min | GR_ANX_DEC |
| **Behaviors affecting satisfaction level** | | |
| Satisfaction increase rate when giving grooming | + 0.05/min | GG_SAT_INC |
| Satisfaction increase rate when receiving grooming | + 0.10/min | GR_SAT_INC |
| Default satisfaction decrease rate | - 0.02/min | DEF_SAT_DEC |
