# Supplementary material for: The EMO-Model: An Agent-Based Model of Primate Social Behavior Regulated by Two Emotional Dimensions, Anxiety-FEAR and Satisfaction-LIKE
Source: PLoS One. 2014 Feb 4;9(2):e87955. doi: 10.1371/journal.pone.0087955 (PMC3913693; doi:10.1371/journal.pone.0087955)
Supplement: Text S1 — Parameterization of arousal. (DOC) [file pone.0087955.s006.doc]

**Parameterization of arousal**

To implement arousal in our model, we constructed a measure based on an array of empirical heart rate (HR) and scratching rate (SR) data from different social contexts (e.g. baseline, post-conflict, grooming). The average baseline HR in macaques described in the literature was 141.1 bpm [1–4] and the average baseline SR was 0.33 bouts/min [5–12]. We further extracted from the empirical literature the average changes in HR and SR in different social contexts, as well as the maximum and minimum rates described in the literature on macaques. These are summarized in Table 1 (second and third column).

To transform these data into an arousal measure to be used in our model we first fitted a linear line through the heart rate (HR) and scratching rate (SR) data. For this, we used the average HR and SR of those social contexts for which both, HR and SR data were available, namely 'Baseline', 'Proximity dominant' and 'Post-conflict victim' (see contexts marked with * in Table 1). Fitting a linear regression model through the origin (minimum heart rate and scratching rate) yielded HR = 127.8 + 38.19 * SR. Using this linear model, we predicted the HR for those social contexts where only SR data were available (see measure indicated in bold type in Table 1). Finally, we converted the empirical and predicted HR data onto a scale ranging from 0 to 1 using the function Arousal level = (HR - 127.8) / 149 (5th column in Table 1). Predicted HR data were used whenever empirical HR data where lacking. Only for 'Proximity dominant' we chose the maximum of predicted and empirical HR to define the maximum arousal increase in this social context (see below). The minimum SR was assumed to be 0.0. This scaled measure was then used as the level of arousal in our model. Thus, in our model arousal level was scaled between 0 (inactive) and 1 (maximum stimulation).

The instantaneous impact of 'Attack received' and 'Attack given' on arousal were based on empirical data (see Table 1). The impact of other point behaviors on arousal was chosen arbitrarily, assuming that receiving an attack has a higher impact than giving or perceiving an attack or receiving an aggressive signal. We assumed, that upon several aggressive events arousal gets increased per event and may build up quickly. We further assumed that aggressive behaviors may increase arousal levels up to the maximum possible arousal level of 1.0 (MAX_AR_LIMIT), while submissive and affiliative signals may decrease increased arousal levels towards the baseline level of 0.09 (DEF_AR_LIMIT), but not below (see Table 2).

The average default decrease rate of arousal was estimated using an array of time series data of heart rate and scratching rate after a conflict (without post-conflict grooming) [1,9,10,13]. After transforming these data to our model arousal measure (see above), we fitted a linear model through each of four datasets of time and (converted) arousal data and averaged the obtained arousal decrease rates. This average arousal decrease rate of 0.02/min was then used as default arousal decrease rate in our model (DEF_AR_DEC). We assumed that the default increase rate is similar to the default decrease rate and also set it to 0.02/min (DEF_AR_INC).

Whenever a dominant individual is perceived in proximity, arousal slowly increases up to a context-specific maximum arousal level (limit value) of 0.12 (PD_AR_LIMIT). The maximum arousal level for this social context was based on empirical data (see Table 1). As arousal increase rate for this social context we arbitrarily chose the same rate as for the default arousal increase rate of 0.02/min (PD_AR_INC). When grooming is given or received, arousal may decrease even below baseline levels down to context-specific minimum values (limit values) of 0.04 (GG_AR_LIMIT) or 0.03 (GR_AR_LIMIT), respectively, but not below. GG_AR_LIMIT and GR_AR_LIMIT were based on empirical data (see Table 1). Note, that for GG_AR_LIMIT we assumed that giving grooming is similarly relaxing as contact sitting (see Table 1). When ego is giving grooming, we assumed that its arousal decreases with a rate similar to the default arousal decrease rate of 0.02/min (GG_AR_DEC). When receiving grooming, arousal is expected to decrease faster than the default arousal decrease rate. The arousal decrease rate during receiving grooming was estimated similarly to the default arousal decrease rate, using an array of time series data of heart rate and scratching rate after a conflict with post-conflict grooming [1,10,13]. After transforming these data to our model arousal measure (see above), we fitted a linear model through each of three datasets of time and (converted) arousal data and averaged the obtained arousal decrease rates. This average arousal decrease rate of 0.04/min was then used as arousal decrease rate during receiving grooming in our model (GR_AR_DEC) (see Table 2).

**Table 1: Arousal levels in various social contexts.**

| **Social context** | **Change (and level) of heart rate in bpm** | **Change (and level) of scratching rate in bouts/min** | **Predicted change (and level) of heart rate in bpm** | **Arousal level in the model** | **Parameter name in the model** | **References empirical heart rate data** | **References empirical scratching rate data** |
| --- | --- | --- | --- | --- | --- | --- | --- |
| Maximum arousal | +135.7 (276.8) |  |  | 1.0 | MAX_AR_LIMIT | [2,3] |  |
| Acute stress peak | **+91.6 (232.7)** |  |  | 0.70 (+0.61) |  | [2,3] |  |
| Agonism | **+25.7 (166.8)** |  |  | 0.26 (+0.27) |  | [1] |  |
| Post-conflict (victim)* | **+12 (153.1)** | +0.31 (0.64) |  | 0.17 (+0.08) | AR_AR_INC | [13] | [6,7,9,10] |
| Post-conflict (aggressor) |  | +0.16 (0.49) | **+5.4 (146.5)** | 0.13 (+0.04) | AG_AR_INC |  | [5,7] |
| Proximity dominant* | +3.4 (144.5) | +0.15 (0.48) | **+5.0 (146.1)** | 0.12 (+0.03) | PD_AR_LIMIT | [14] | [8] |
| Baseline* | **±0.0 (141.1)** | ± 0.0 (0.33) |  | 0.09 | DEF_AR_LIMIT | [1–4] | [5–12] |
| Physical contact  (Grooming given) |  | -0.17 (0.16) | **-7.2 (133.9)** | 0.04 | GG_AR_LIMIT |  | [8] |
| Grooming received | **-9.5 (131.6)** |  |  | 0.03 | GR_AR_LIMIT | [1,14] |  |
| Minimum arousal level* | **-13.3 (127.8)** | -0.33 (0.00) |  | 0.0 |  | [3] |  |

Column two and three summarize how heart and scratching rate differ from baseline levels in different social contexts (first column) reported in the literature (last two columns). In brackets, we also mention the respective expected level of heart and scratching rate assuming baseline levels of 141.1 bpm (heart rate) and 0.33 (scratching rate). Values of those social contexts where heart and scratching rate data were available (marked with *) were used to obtain a linear model to predict heart rate from scratching rate (third column). Those heart rate data (empirical or predicted) that were used to obtain the model arousal measure (4th column) are indicated in bold type.

**Table 2: Effect of social behaviors on arousal levels.**

| **Behavior** | **Change of arousal level in the model** | **Parameter name** | **Maximum / minimum arousal level caused by behavior** | **References empirical data** |
| --- | --- | --- | --- | --- |
| **Point behaviors** | | | | |
| Escalated fight observed | +0.04 | EFO_AR_INC | MAX_AR_LIMIT |  |
| Aggressive signal received | +0.04 | ASR_AR_INC | MAX_AR_LIMIT |  |
| Attack given | +0.04 | AG_AR_INC | MAX_AR_LIMIT |  |
| Attack received | +0.08 | AR_AR_INC | MAX_AR_LIMIT |  |
| Affiliative signal received | -0.04 | AS_AR_DEC | DEF_AR_LIMIT |  |
| Submissive signal received | -0.04 | SS_AR_DEC | DEF_AR_LIMIT |  |
| **Duration behaviors** | | | | |
| Default decrease | - 0.02 / min | DEF_AR_DEC | DEF_AR_LIMIT | [1,9,10,13] |
| Default increase | + 0.02 / min | DEF_AR_INC | DEF_AR_LIMIT |  |
| Proximity of dominant | + 0.02 / min | PD_AR_INC | PD_AR_LIMIT |  |
| Grooming given | - 0.02 / min | GG_AR_DEC | GG_AR_LIMIT |  |
| Grooming received | - 0.04 / min | GR_AR_DEC | GR_AR_LIMIT | [1,10,13] |

**References**

1. Boccia ML, Reite M, Laudenslager M (1989) On the physiology of grooming in a pigtail macaque. Physiol Behav 45: 667–670.

2. Manuck S, Kaplan J, Adams M, Clarkson T (1989) Behaviorally elicited heart rate reactivity and atherosclerosis in female cynomolgus monkeys (Macaca fascicularis). Psychosom Med 51: 306–318.

3. Manuck S, Kaplan J, Clarkson T (1983) Behaviorally induced heart rate reactivity and atherosclerosis in cynomolgus monkeys. Psychosom Med 45: 95–108.

4. Watson SL, Shively CA, Kaplan JR, Line SW (1998) Effects of chronic social separation on cardiovascular disease risk factors in female cynomolgus monkeys. Atherosclerosis 137: 259–266. doi:10.1016/S0021-9150(97)00277-3.

5. Aureli F (1997) Post-conflict anxiety in nonhuman primates: The mediating role of emotion in conflict resolution. Aggress Behav 23: 315–328.

6. Aureli F, Van Schaik CP (1991) Post-conflict behaviour in long-tailed macaques (Macaca fascicularis): II. Coping with the uncertainty. Ethology 89: 101–114.

7. Schino G, Rosati L, Geminiani S, Aureli F (2007) Post-Conflict Anxiety in Japanese Macaques (Macaca fuscata): Aggressor’s and Victim’s Perspectives. Ethology 113: 1081–1088. doi:10.1111/j.1439-0310.2007.01407.x.

8. Pavani S, Maestripieri D, Schino G, Turillazzi PG, Scucchi S (1991) Factors influencing scratching behaviour in long-tailed macaques (Macaca fascicularis). Folia Primatol 57: 34–38.

9. Kutsukake N, Castles DL (2001) Reconciliation and variation in post-conflict stress in Japanese macaques (Macaca fuscata fuscata): testing the integrated hypothesis. Anim Cogn 4: 259–268. doi:10.1007/s10071-001-0119-2.

10. Aureli F, Van Schaik CP, Van Hooff JARAM (1989) Functional aspects of reconciliation among captive long-tailed macaques (Macaca fascicularis). Am J Primatol 19: 39–51. doi:10.1002/ajp.1350190105.

11. Aureli F (1992) Post-conflict behaviour among wild long-tailed macaques (Macaca fascicularis). Behav Ecol Sociobiol 31. doi:10.1007/BF00177773.

12. Aureli F, Yates K (2010) Distress prevention by grooming others in crested black macaques. Biol Lett 6: 27–29. doi:10.1098/rsbl.2009.0513.

13. Aureli F, Smucny DA (2000) The role of emotion in conflict and conflict resolution. Nat Confl Resolut: 199–224.

14. Aureli F, Preston SD, de Waal FBM (1999) Heart Rate Responses to Social Interactions in Free-Moving Rhesus Macaques (Macaca mulatta): A Pilot Study,. J Comp Psychol 113: 59–65.
