# Supplementary material for: The EMO-Model: An Agent-Based Model of Primate Social Behavior Regulated by Two Emotional Dimensions, Anxiety-FEAR and Satisfaction-LIKE
Source: PLoS One. 2014 Feb 4;9(2):e87955. doi: 10.1371/journal.pone.0087955 (PMC3913693; doi:10.1371/journal.pone.0087955)
Supplement: Text S2 — General behavioral probabilities. (DOC) [file pone.0087955.s007.doc]

**General behavioral probabilities**

Ego's arousal level (myAROUSAL) positively affects the probabilities for active behaviors during action selection, i.e. any behavior except resting (see Table 1). GG_PROB, AFS_PROB, APP_PROB, ATT_PROB, AGS_PROB, LE_PROB, SS_PROB, AV_PROB, RNDW_PROB and REST_PROB are fixed parameters, which were used to tune the relative probabilities between all behaviors such that the resulting average behavioral frequencies represented empirical observations. The values for these parameters are given in Table 2.

The general affiliation probability (myAFF_PROB_ij_), which affects the probabilities of grooming, affiliative signals and approach (see Table 1), is calculated as follows:

.

Here, i is the actor and j is the (potential) receiver of the affiliative behavior and LIKE_ij_ is the LIKE attitude directed from individual i to j. AFF_0_ is a constant parameter, which determines the maximum probability to execute affiliative behavior when the actor has no intrinsic motivation to affiliate (myAFF_MOT_i_ = 0, see solid lines at LIKE=1 in Figure 1A). AFF_0_ is set arbitrarily to 0.1. Increased intrinsic motivation to affiliate (myAFF_MOT_i_>0) may increase the affiliation probability up to 1.0 (see dashed and dotted lines at LIKE=1 in Figure 1A). LPS is the degree to which LIKE attitudes are of importance for the affiliation probability. At LPS=0, the level of LIKE_ij_ has no effect on the affiliation probability (see first panel of Figure 1A). LPS describes the degree to which the maximum affiliation probability (at LIKE=1) decreases with decreasing LIKE_ij_ (see steeper slopes of the lines at increased LPS in Figure 1A). Thus, in our model, myAFF_PROB_ij_ increases with increased LIKE_ij_ (given that LPS>0) and with increased myAFF_MOT_i_.

The intrinsic affiliation motivation of individual i depends on its satisfaction and anxiety and is calculated as follows:

.

Here, SW and AW are fixed weighting factors, which determine the relative importance of satisfaction and anxiety, respectively, for the intrinsic affiliation motivation. Intrinsic affiliation motivation is high when ego has a low level of satisfaction and a high level of anxiety. SW is set arbitrarily to 0.1 and AW is set arbitrarily to 0.9. Thus, we assumed, that high anxiety is affecting the intrinsic affiliation motivation to a higher degree than low satisfaction.

The general aggression probability (myAGG_PROB_ij_), which affects the probabilities of attacking and aggressive signals (see Table 1), is calculated as follows:

.

Here, i is the actor and j is the (potential) receiver of the aggressive behavior and FEAR_ij_ is the FEAR attitude, i.e. the difference between the dominance strength of individual j and individual i. myAGG_PROB_ij_ is a decreasing sigmoid function of FEAR_ij_ (see first panel of Figure 1B). AGG_0_ is a constant parameter, which determines the baseline maximum probability to execute aggressive behavior at minimum FEAR_ij_ and minimum myANXIETY_i_ (see solid line, myANXIETY_i_=0, in first panel of Figure 1B at FEAR_ij_=-1). AGG_0_ is set arbitrarily to 0.1. The maximum probability to execute aggressive behavior at minimum FEAR_ij_ (FEAR_ij_=-1) is increased with increased myANXIETY_i_ (see dashed and dotted line at FEAR_ij_=-1 in the first panel of Figure 1B).

The slope and the location of the inflection point of the sigmoid function depend on the level of myANXIETY_i_ and are determined, via the parameters AGG_IPD_ and AGG_SI_, by the function myRISK_ij_(FEAR_ij_,myANXIETY_i_) as follows:

.

Here, AGG_IPD_ is a fixed parameter, which describes the maximum decrease of the inflection point of the sigmoid function relative to the baseline sigmoid function at myANXIETY_i_=0. AGG_IPD_ is set arbitrarily to 0.5, thus at maximum myANXIETY_i_ (myANXIETY_i_=1), the inflection point shifts from 0 to -0.5 (compare the inflection points of the solid and dotted line in the first panel of Figure 1B). The fixed parameter η describes the baseline slope of the sigmoid function and is set to 6. Note, that in the baseline situation (myANXIETY_i_=0), myAGG_PROB_ij_/AGG_0_ would actually describe individual i's chance of winning an escalated fight from individual j. Thus, η here is the same as in the win chance function w_ij_. Finally, AGG_SI_ is a fixed parameter, which determines the maximum increase of the slope of the sigmoid function relative to the baseline sigmoid function at myANXIETY_i_=0. AGG_SI_ is set arbitrarily to 1. Note, that the slope of myAGG_PROB_ij_ is also affected by (AGG_0_ + (1-AGG_0_)*myANXIETY­_i_). Taken together, this results in a slope of the sigmoid function at myANXIETY_i_=1, which is twenty times as steep compared to myANXIETY_i_=0 (compare slope of the solid and dotted line in the first panel of Figure 1B).

In sum, higher levels of anxiety result in a more conservative (or risk avoiding) probability of aggression concerning the FEAR attitudes directed to the potential receivers of aggression. On the one hand, when the FEAR attitude towards the opponent is very low (i.e. the opponent is much lower-ranking than ego) aggression probability increases with increased anxiety. On the other hand, the inflection point of the sigmoid function decreases. Thus, at low anxiety aggression probability changes from high to low around FEAR=0 (i.e. similar-ranking opponent), while at high anxiety it already changes from high to low around FEAR=0.5 (i.e. somewhat lower-ranking opponent)(compare solid, dashed and dotted line in the first panel of Figure 1B).

The general submission probability (mySUB_PROB_ij_), which affects the probabilities of leaving and submissive signals (see Table 1), is calculated as follows:

.

Here, SE is a fixed parameter, which increases the effect of FEAR at higher FEAR (see second panel in Figure 1B). SE was arbitrarily set to 4. SUB_0_ is a fixed parameter, which describes the maximum submission probability when myANXIETY_i_=0 (see solid line at FEAR=+1 in the second panel in Figure 1B). SUB_0_ was set arbitrarily to 0.1. The maximum submission probability at maximum FEAR_ij_ is increased with increased myANXIETY_i_ (see dashed and dotted line at FEAR=+1 in the second panel in Figure 1B). Moreover, submission was by definition only directed at individuals towards which ego directed a positive FEAR attitude, i.e. individuals that were higher in rank than ego.

The general avoidance probability (myAV_PROB_ij_), which affects the probability of avoidance behavior (see Table 1), is calculated as follows:

.

Here, AE is a fixed parameter similarly to SE in the calculation of the submission probability above. However, AE was set to a higher value (AE=16), i.e. the effect of FEAR was even stronger depending on the value of FEAR. Thus, the avoidance probability was calculated very similarly to the submission probability, except that it only increased substantially at very high FEAR (see third panel in Figure 1B).

The probability to perform scanning behavior increases with the individual's current arousal level as follows:

.

Thus, highly aroused individuals employ scanning, while non-aroused individuals do not. For moderate arousal levels, just above the baseline arousal level and just below the increased arousal after 'Receiving a (single) attack', the scanning probability increases linearly with arousal (see Figure 2). SPS is a fixed parameter, which describes the slope with which scanning probability increases for moderate arousal levels. SPO is a fixed parameter, which describes the offset of the linear increasing scanning probability at moderate arousal levels. SPS was set to 18.0 and SPO was set to 1.8, which resulted in average percentages of time spent scanning that were reasonable for macaques.

**
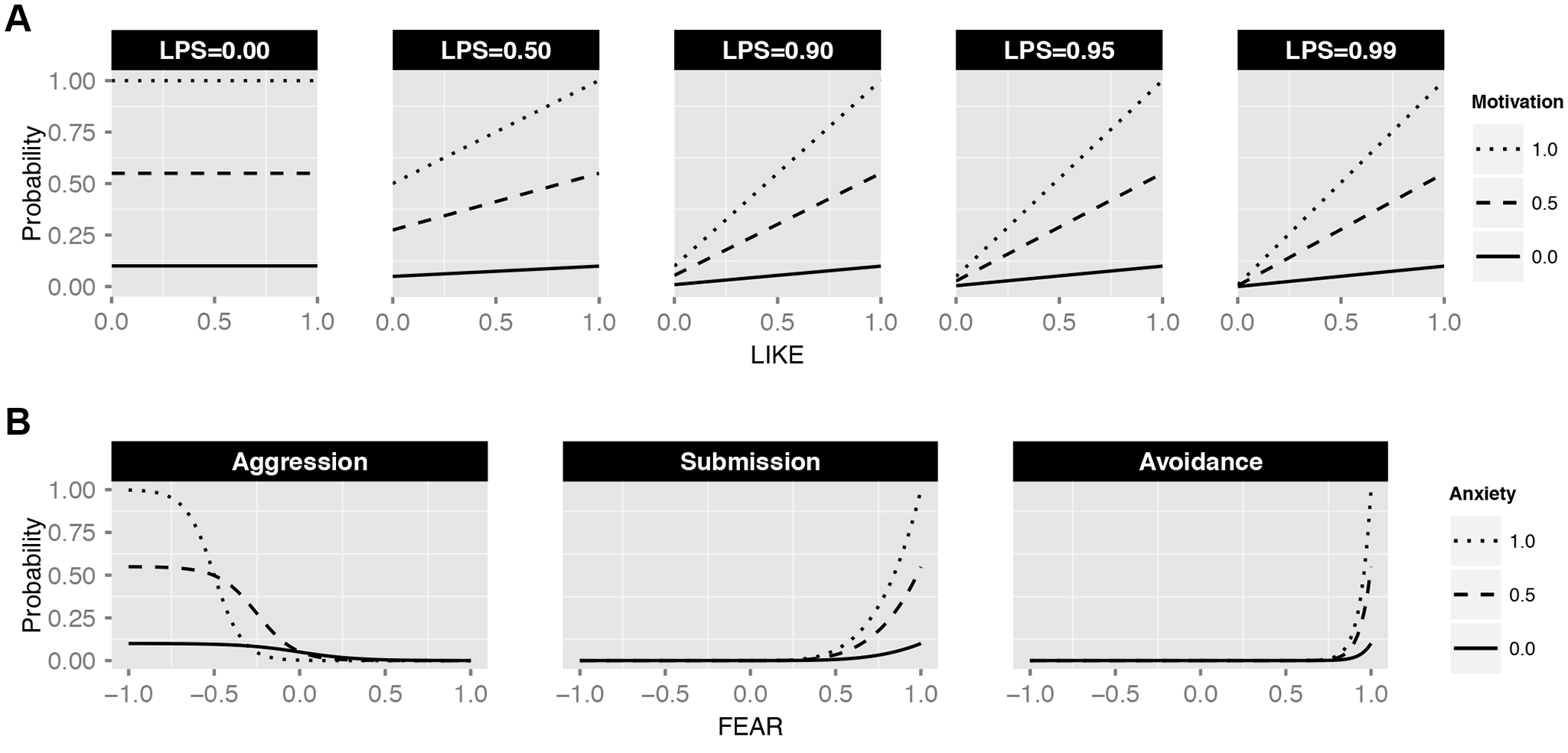
**

**Figure 1: Behavioral probability functions.** The upper part of this figure (**A**) shows the general probability for affiliative behavior as a function of the LIKE attitude of ego towards the potential partner (x-axis), depending on the level of an individual's intrinsic motivation to perform affiliative behavior (dotted line: high motivation, dashed line: intermediate motivation, solid line: no intrinsic motivation) and on the setting of LPS (see panels). The internal motivation is calculated based on ego's level of anxiety and satisfaction (see text and equation for myAFF_MOT). Higher LPS results in lower affiliation probabilities for potential partners towards whom ego assigns a low LIKE attitude. Thus, with higher LPS ego becomes more selective and prefers high-LIKE partners relatively more than low-LIKE partners. The lower part of this figure (**B**) shows the probability for agonistic behavior as a function of the FEAR attitude of ego towards the potential partner (x-axis), depending on the level of ego's anxiety (dotted line: high anxiety, dashed line: intermediate anxiety, solid line: no anxiety). The panel shows the specific behavioral probabilities for aggression, submission and avoidance.


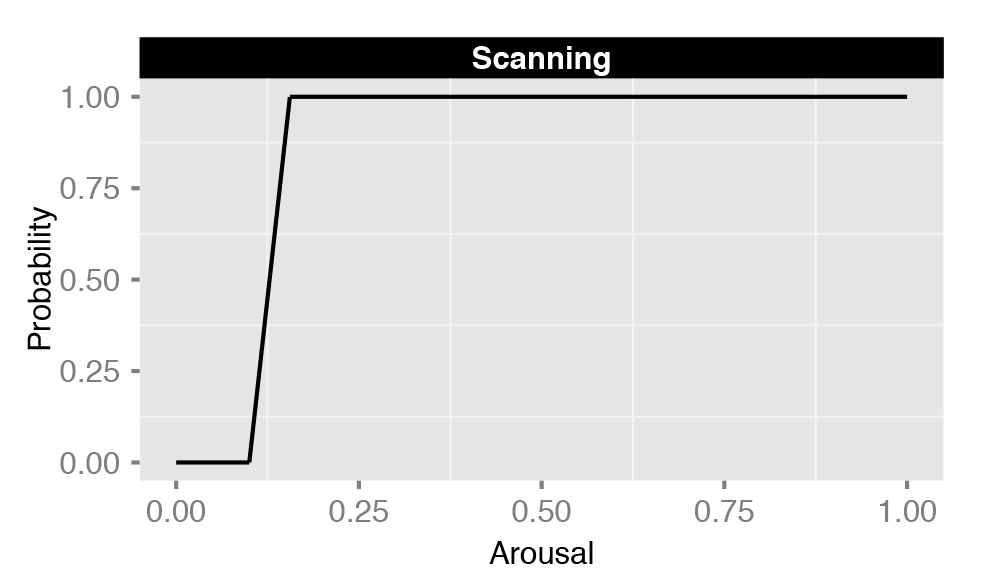


**Figure 2: Scanning probability.** This figure shows the probability to employ scanning behavior as a function of ego's arousal level (x-axis).

**Table 1: Behavioral probabilities for individual i.**

| **Behavior** | **Probability** |
| --- | --- |
| **Affiliative behaviors towards individual j** | |
| Grooming | myAROUSAL_i_ * GG_PROB * myAFF_PROB_ij_ |
| Affiliative signal | myAROUSAL_i_ * AFS_PROB * myAFF_PROB_ij_ |
| Approach | myAROUSAL_i_ * APP_PROB * myAFF_PROB_ij_ |
| **Aggressive behavior towards individual j** | |
| Attack | myAROUSAL_i_ *ATT_PROB * myAGG_PROB_ij_ |
| Aggressive signal | myAROUSAL_i_ * AGS_PROB * myAGG_PROB_ij_ |
| **Submissve behavior towards individual j** | |
| Leave | myAROUSAL_i_ * LE_PROB * mySUB_PROB_ij_ |
| Submissive signal | myAROUSAL_i_ * SS_PROB * mySUB_PROB_ij_ |
| Avoid | myAROUSAL_i_ * AV_PROB * myAV_PROB_ij_ |
| **Non-directed behavior** | |
| Random walk | myAROUSAL_i_ * RNDW_PROB |
| Rest | REST_PROB |

**Table 2: Action selection parameters.**

| **Action selection parameter name** | **Description** | **Value** |
| --- | --- | --- |
| AFF_0_ | Baseline affiliation probability when intrinsic affiliation motivation is 0 | 0.1 |
| SW | Weight of satisfaction for intrinsic affiliation motivation | 0.1 |
| AW | Weight of anxiety for intrinsic affiliation motivation | 0.9 |
| AGG_0_ | Baseline maximum aggression probability when FEAR_ij_=-1 and myANXIETY_i_=0 | 0.1 |
| AGG_IPD_ | Maximum shift of inflection point of aggression probability | 0.5 |
| AGG_SI_ | Maximum increase of slope of aggression probability | 1.0 |
| η | Default slope of aggression probability and win chance w_ij_ | 6 |
| SUB_0_ | Baseline maximum submission or avoidance probability when myANXIETY_i_=0 | 0.1 |
| SE | Parabolic effect of FEAR on submission probability | 4 |
| AE | Parabolic effect of FEAR on submission probability | 16 |
| GG_PROB | Relative probability factor for grooming | 10 |
| AFS_PROB | Relative probability factor for affiliative signal | 50 |
| APP_PROB | Relative probability factor for approach | 100 |
| ATT_PROB | Relative probability factor for attack | 10 |
| AGS_PROB | Relative probability factor for aggressive signal | 50 |
| LE_PROB | Relative probability factor for leaving | 40 |
| SS_PROB | Relative probability factor for submissive signal | 50 |
| AV_PROB | Relative probability factor for avoidance | 100 |
| RNDW_PROB | Relative probability factor for random walk | 0.1 |
| REST_PROB | Relative probability factor for resting | 0.2 |
