# Supplementary material for: The EMO-Model: An Agent-Based Model of Primate Social Behavior Regulated by Two Emotional Dimensions, Anxiety-FEAR and Satisfaction-LIKE
Source: PLoS One. 2014 Feb 4;9(2):e87955. doi: 10.1371/journal.pone.0087955 (PMC3913693; doi:10.1371/journal.pone.0087955)
Supplement: Text S3 — Technical details on movement in bouts. (DOC) [file pone.0087955.s008.doc]

**Technical details on movement in bouts**

Movement behavior in the EMO-model takes time and is implemented as movement bouts. Individuals in our model move with a constant speed of 0.6 m/s, which is reasonable for macaques [1]. During such a movement bout, movement is executed step by step. After starting a movement bout, ego is activated each 3 SECONDS to execute the movement it was to perform during this time interval and to decide whether movement is to be continued. During such a movement 'step' individuals move 1.8m. Whenever ego had decided to move towards or away from a certain individual at the start of a movement bout, this goal stays consistent during the whole movement bout. Moreover, if ego approaches or avoids an individual that itself is moving, after each step ego adjusts its movement direction towards (or away from) the individual's updated position. This step-by-step implementation allows individuals to visually and physically “follow” another moving individual.

The parameter STOP_CHANCE describes the probability to end the current movement bout. STOP_CHANCE was set to 0.1, which resulted in an average movement bout distance of around 6.5m that are reasonable for macaques [1]. Whenever an approached individual is reached, the movement bout is ended. An individual could be approached up to 0.5m from the x-y coordinates that defined its spatial location, to account for the physical space an animal requires, i.e. individuals cannot be "on top of each other", but move in a 2D-field and take up space. Whenever an individual that is to be approached is closer to ego than one movement step (1.8m), ego moves only the actual distance towards the individual and then this movement step also takes only the respective amount of time.

**References**

1. Beisner BA, Isbell LA (2009) Movement ecology in a captive environment: the effects of ground substrate on movement paths of captive rhesus macaques, Macaca mulatta. Anim Behav 78: 1269–1277. doi:10.1016/j.anbehav.2009.09.004.
